# Supplementary material for: Perish the thawed? EDTA reduces DNA degradation during extraction from frozen tissue
Source: PLoS One. 2025 Jun 3;20(6):e0321872. doi: 10.1371/journal.pone.0321872 (PMC12132941; doi:10.1371/journal.pone.0321872)
Supplement: S1 Fig — DNA was extracted from frozen tissue samples collected from each of 10 individuals of 10 marine species (five marine fishes and five marine invertebrates) that were thawed in EDTA (250 mM, pH 10; lanes 1–10) or ethanol (95%; lanes 11–20) overnight at 4°C or extracted directly from frozen tissues without subsequent liquid preservative treatment (lanes 21–30). Lanes marked with an L contain 0.66 μL of Quick Load Purple 1 kb Plus DNA Ladder (100 μg/mL; New England Biolabs; Ipswich, MA), except for Centropristis striata, for which lanes marked with an L contain 1 μL of Quick Load Purple 1 kb Plus DNA Ladder. Specimens are presented in the same order across all treatments. (PDF) [file pone.0321872.s001.pdf]

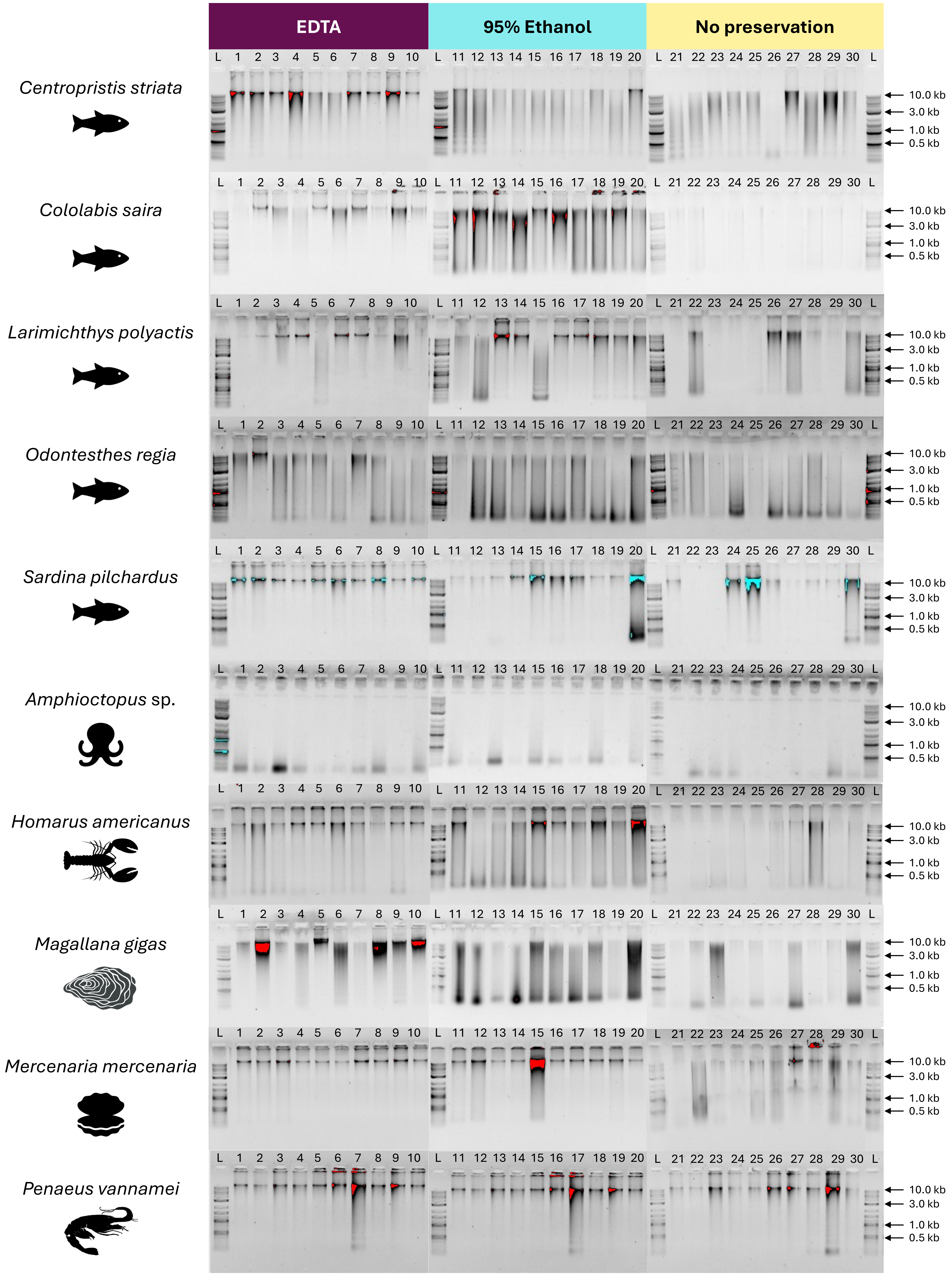
EDTA
95% Ethanol
No preservation
L 1 2 3 4 5 6 7 8 9 10
L 11 12 13 14 15 16 17 18 19 20
L 21 22 23 24 25 26 27 28 29 30 L
Centropristis striata
Cololabis saira
Larimichthys polyactis
Odontesthes regia
Sardina pilchardus
Amphioctopus sp.
Homarus americanus
Magallana gigas
Mercenaria mercenaria
Penaeus vannamei
10.0 kb
3.0 kb
1.0 kb
0.5 kb
