# Supplementary material for: Perish the thawed? EDTA reduces DNA degradation during extraction from frozen tissue
Source: PLoS One. 2025 Jun 3;20(6):e0321872. doi: 10.1371/journal.pone.0321872 (PMC12132941; doi:10.1371/journal.pone.0321872)
Supplement: S2 Fig — DNA was extracted from tissues of six additional marine fish species that were thawed in EDTA (250 mM, pH 10; lanes 1–2) or ethanol (95%; lanes 3–4) overnight at 4°C or extracted directly from frozen tissues without subsequent liquid preservative treatment (lanes 5–6) from two randomly selected specimens of each species. Lanes marked with an L contain 0.66 μL of Quick Load Purple 1 kb Plus DNA Ladder (100 μg/mL; New England Biolabs; Ipswich, MA). Specimens are presented in the same order across all treatments. (PDF) [file pone.0321872.s002.pdf]

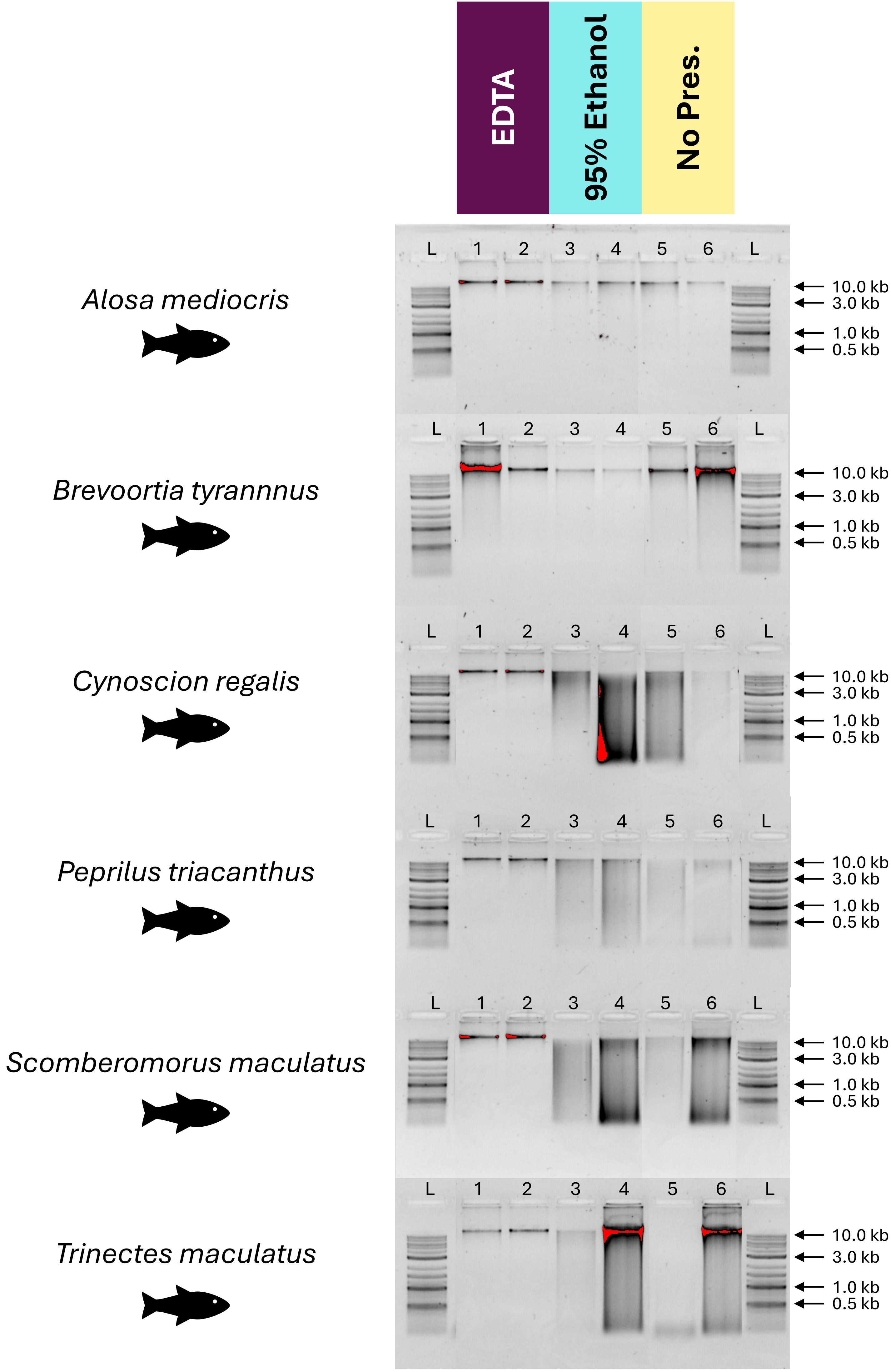

EDTA
95% Ethanol
No Pres.
Alosa mediocris
Brevoortia tyrannnus
Cynoscion regalis
Peprilus triacanthus
Scomberomorus maculatus
Trinectes maculatus
L 1 2 3 4 5 6 L
10.0 kb
3.0 kb
1.0 kb
0.5 kb
