# Supplementary material for: Perish the thawed? EDTA reduces DNA degradation during extraction from frozen tissue
Source: PLoS One. 2025 Jun 3;20(6):e0321872. doi: 10.1371/journal.pone.0321872 (PMC12132941; doi:10.1371/journal.pone.0321872)
Supplement: S3 Fig — DNA was extracted from frozen tissue samples collected from each of 10 individuals of 10 marine species (five marine fishes and five marine invertebrates) that were thawed in EDTA (250 mM, pH 10; lanes 21–30) or ethanol (95%; lanes 11–20) overnight at 4°C or extracted directly from frozen tissues without subsequent liquid preservative treatment (lanes 1–10). Lanes marked with an L contain either 0.33 or 0.66 μL of Quick Load Purple 1 kb Plus DNA Ladder (100 μg/mL; New England Biolabs; Ipswich, MA), except for Centropristis striata, for which lanes marked with an L contain 1 μL of Quick Load Purple 1 kb Plus DNA Ladder. Specimens are presented in the same order across all treatments. Dashes indicate empty lanes. (PDF) [file pone.0321872.s003.pdf]

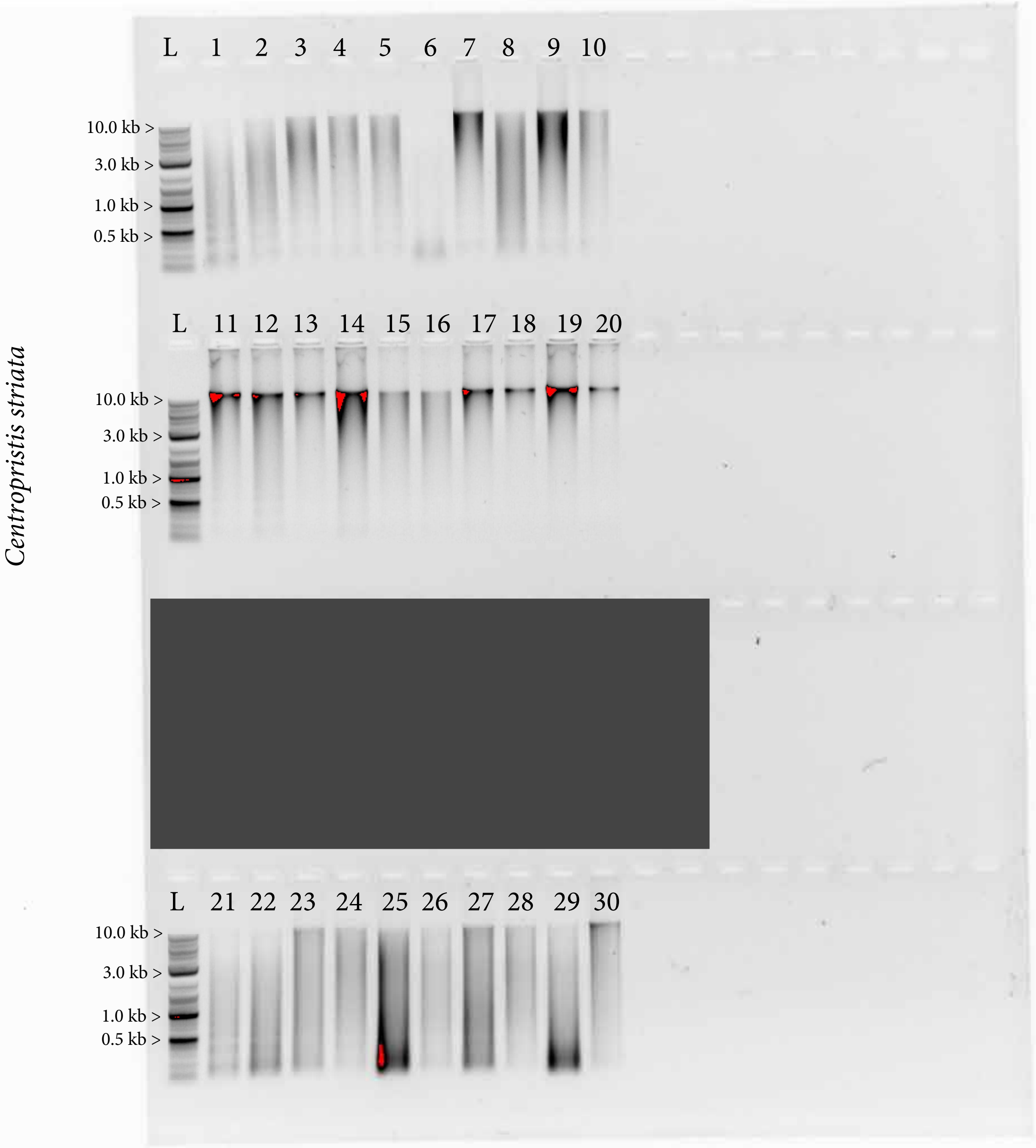

Centropristis striata
L 1 2 3 4 5 6 7 8 9 10
10.0 kb >
3.0 kb >
1.0 kb >
0.5 kb >
L 11 12 13 14 15 16 17 18 19 20
10.0 kb >
3.0 kb >
1.0 kb >
0.5 kb >
L 21 22 23 24 25 26 27 28 29 30
10.0 kb >
3.0 kb >
1.0 kb >
0.5 kb >

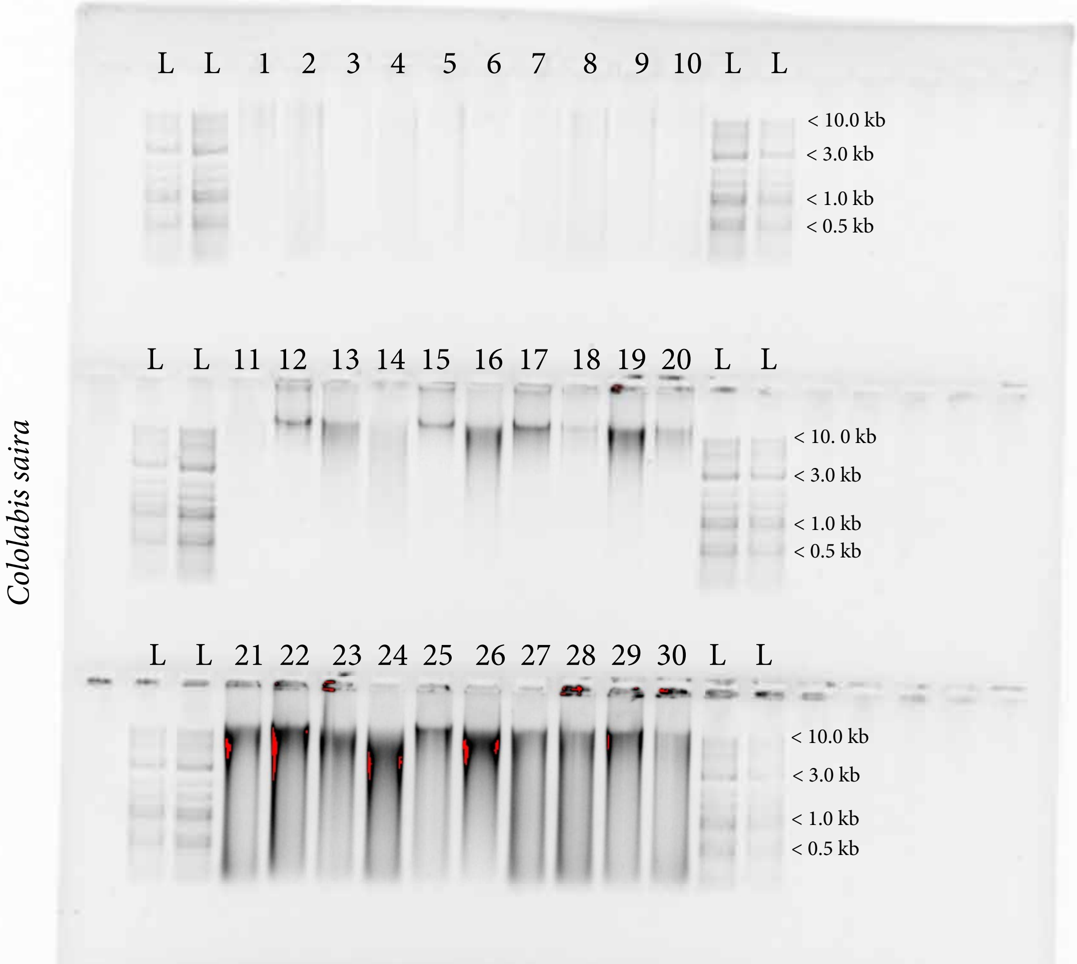
Cololabis saira
L L 1 2 3 4 5 6 7 8 9 10 L L
< 10.0 kb
< 3.0 kb
< 1.0 kb
< 0.5 kb
L L 11 12 13 14 15 16 17 18 19 20 L L
< 10. 0 kb
< 3.0 kb
< 1.0 kb
< 0.5 kb
L L 21 22 23 24 25 26 27 28 29 30 L L
< 10.0 kb
< 3.0 kb
< 1.0 kb
< 0.5 kb

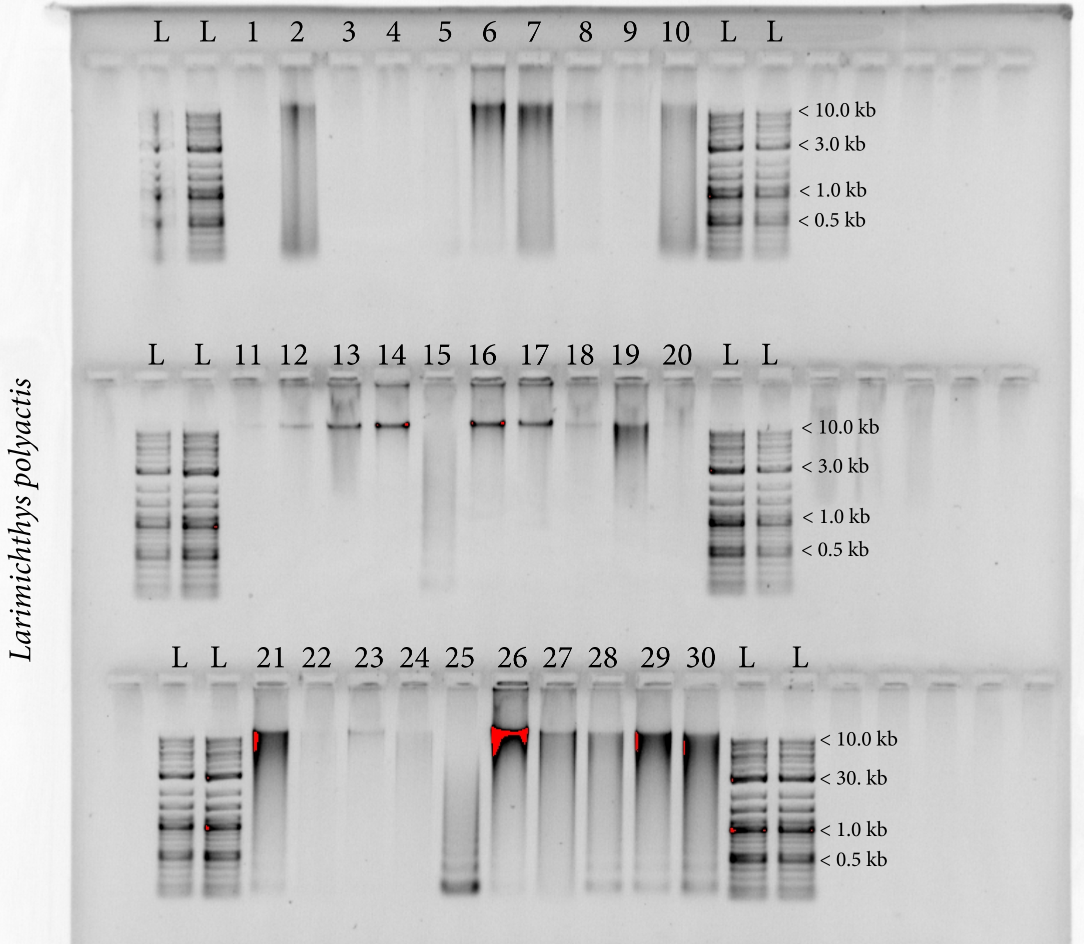
Larimichthys polyactis
L L 1 2 3 4 5 6 7 8 9 10 L L
< 10.0 kb
< 3.0 kb
< 1.0 kb
< 0.5 kb
L L 11 12 13 14 15 16 17 18 19 20 L L
< 10.0 kb
< 3.0 kb
< 1.0 kb
< 0.5 kb
L L 21 22 23 24 25 26 27 28 29 30 L L
< 10.0 kb
< 30. kb
< 1.0 kb
< 0.5 kb

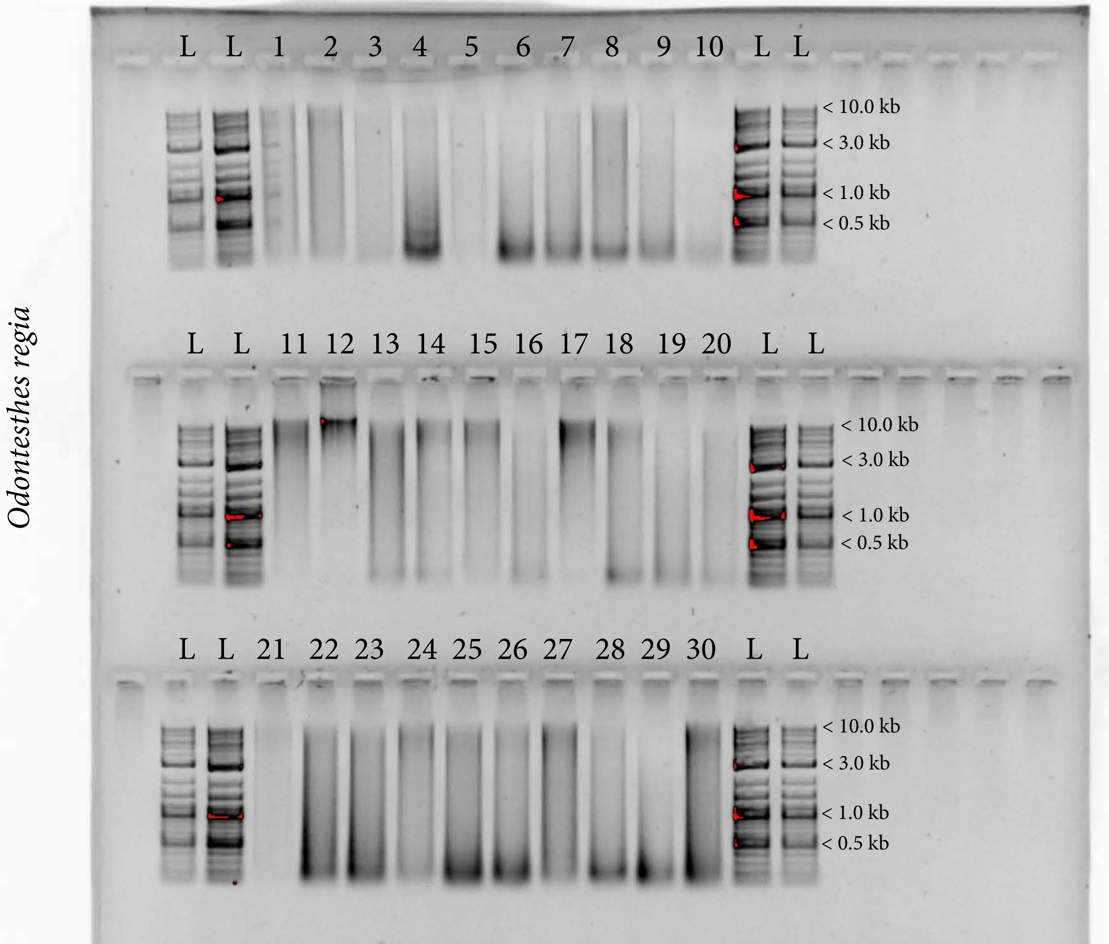
Odontesthes regia
L L 1 2 3 4 5 6 7 8 9 10 L L
< 10.0 kb
< 3.0 kb
< 1.0 kb
< 0.5 kb
L L 11 12 13 14 15 16 17 18 19 20 L L
< 10.0 kb
< 3.0 kb
< 1.0 kb
< 0.5 kb
L L 21 22 23 24 25 26 27 28 29 30 L L
< 10.0 kb
< 3.0 kb
< 1.0 kb
< 0.5 kb

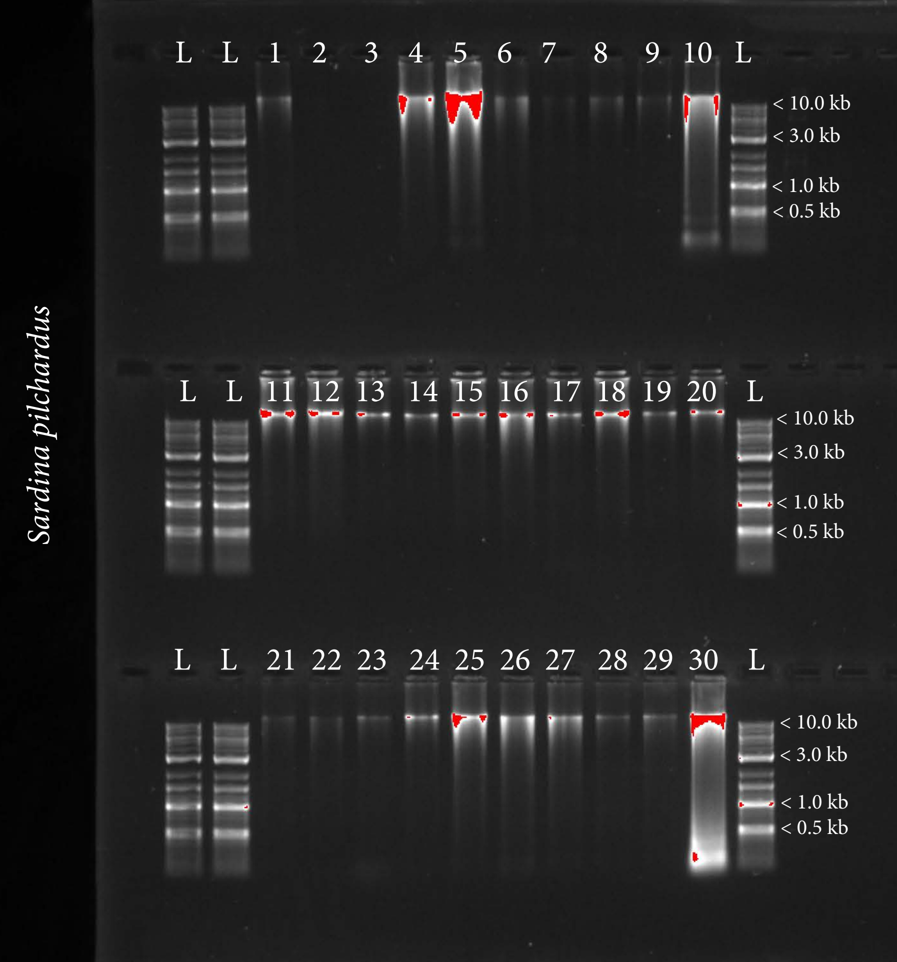
Sardina pilchardus
L L 1 2 3 4 5 6 7 8 9 10 L
< 10.0 kb
< 3.0 kb
< 1.0 kb
< 0.5 kb
L L 11 12 13 14 15 16 17 18 19 20 L
< 10.0 kb
< 3.0 kb
< 1.0 kb
< 0.5 kb
L L 21 22 23 24 25 26 27 28 29 30 L
< 10.0 kb
< 3.0 kb
< 1.0 kb
< 0.5 kb

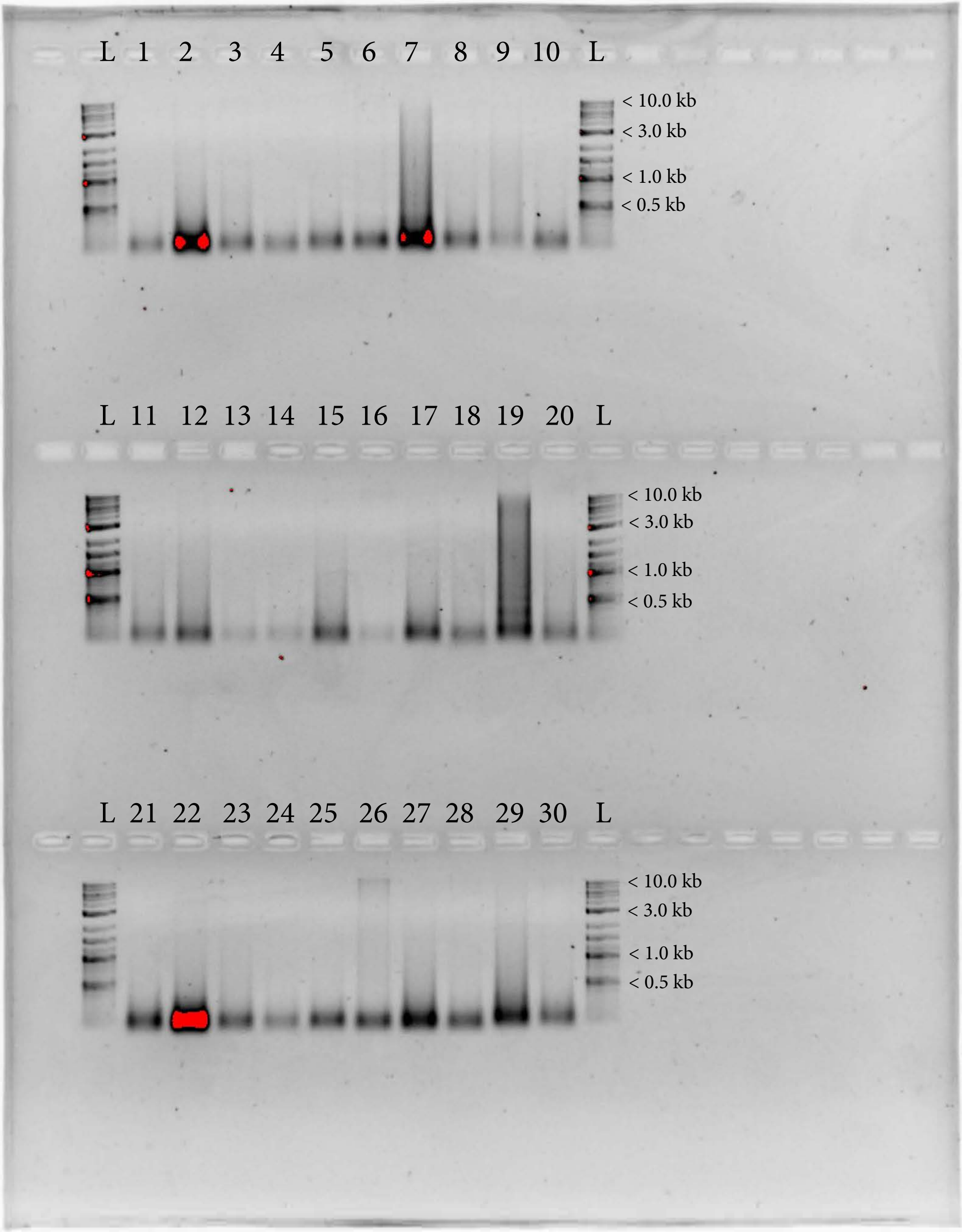

Amphioctopus sp.

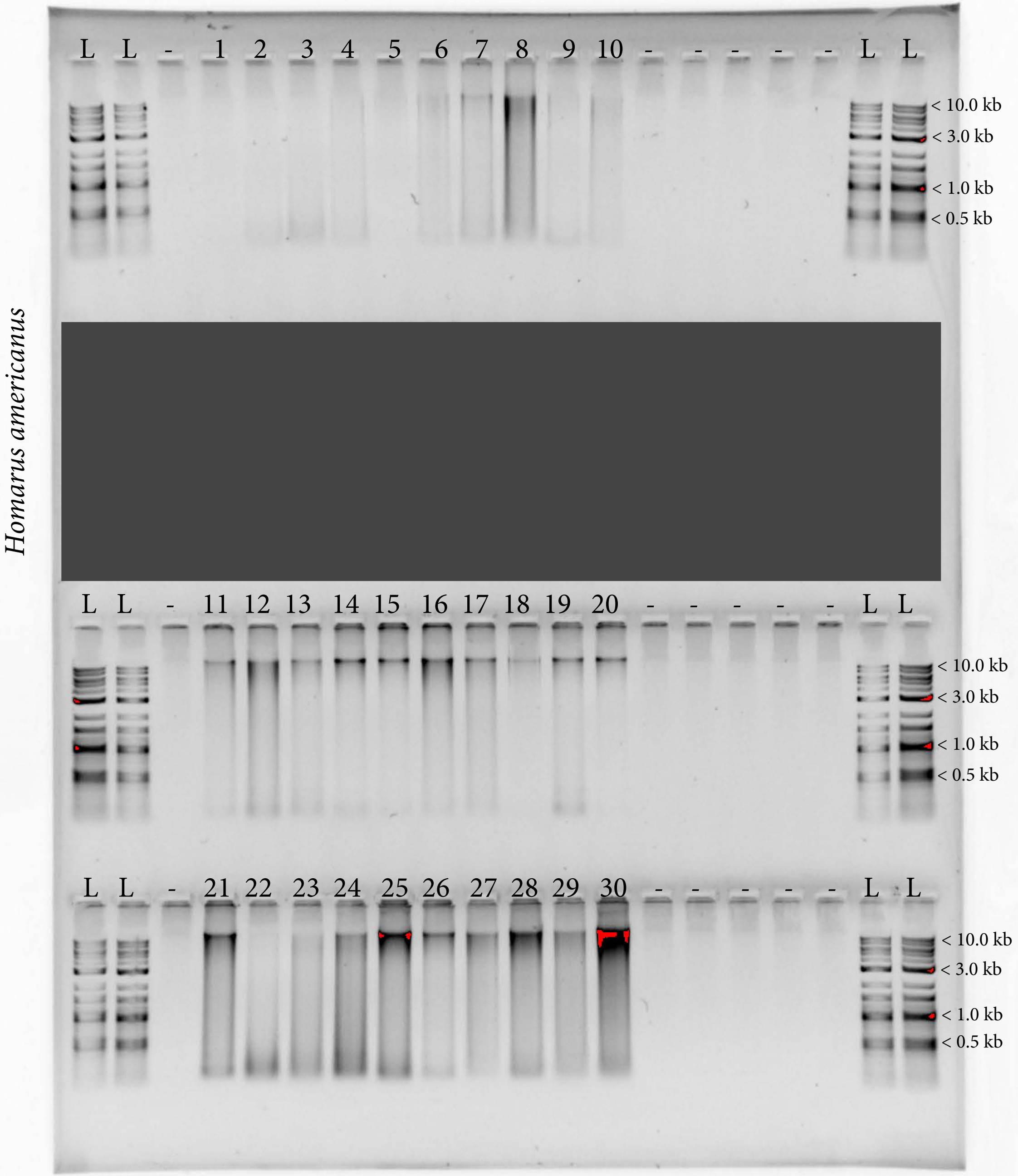

Homarus americanus
L L - 1 2 3 4 5 6 7 8 9 10 - - - - - L L
< 10.0 kb
< 3.0 kb
< 1.0 kb
< 0.5 kb
L L - 11 12 13 14 15 16 17 18 19 20 - - - - - L L
< 10.0 kb
< 3.0 kb
< 1.0 kb
< 0.5 kb
L L - 21 22 23 24 25 26 27 28 29 30 - - - - - L L
< 10.0 kb
< 3.0 kb
< 1.0 kb
< 0.5 kb

Magallana gigas

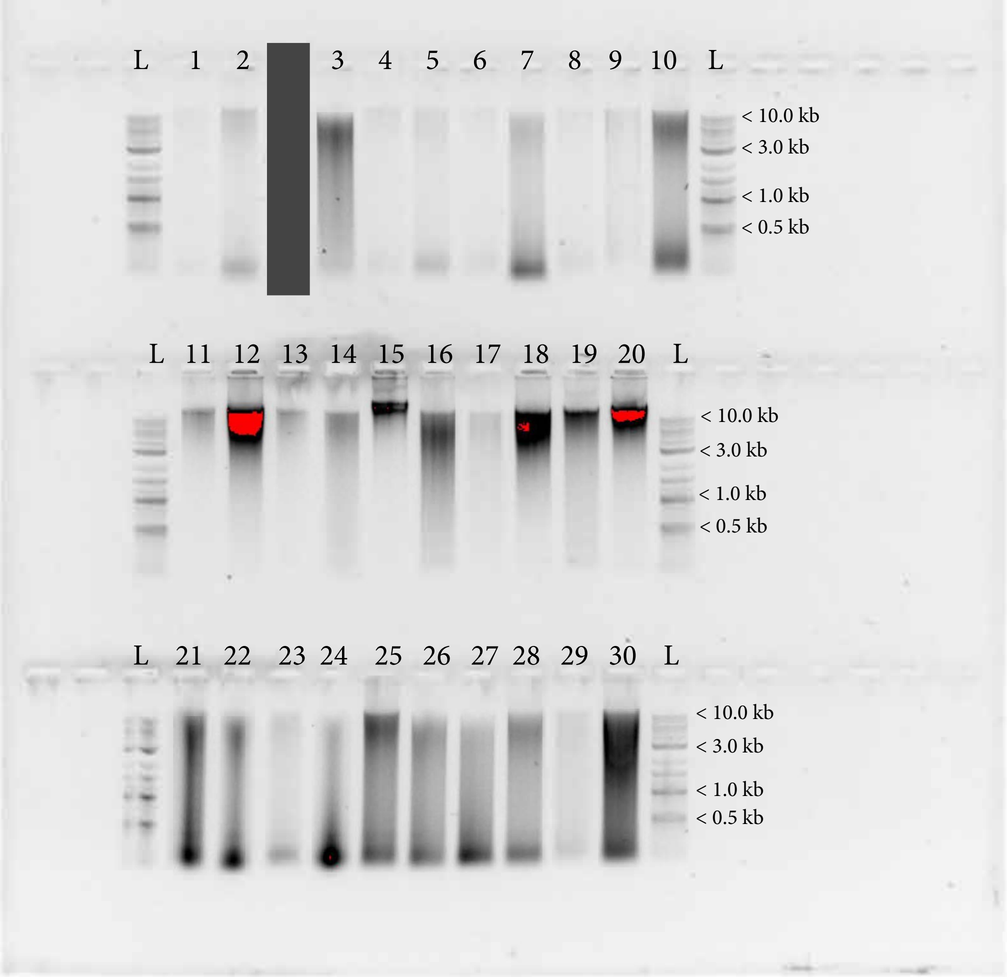

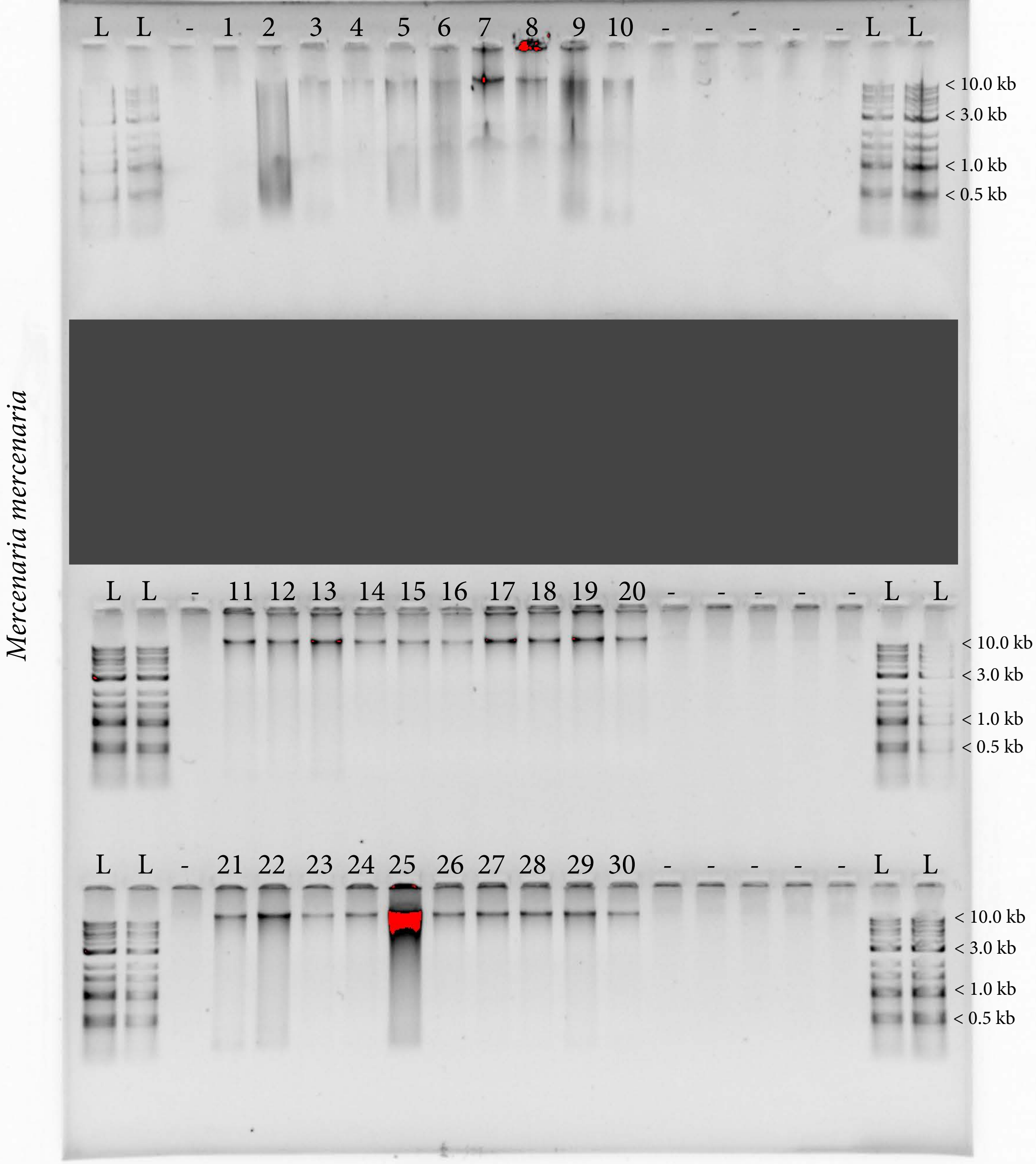
Mercenaria mercenaria
L L - 1 2 3 4 5 6 7 8 9 10 - - - - - L L
< 10.0 kb
< 3.0 kb
< 1.0 kb
< 0.5 kb
L L - 11 12 13 14 15 16 17 18 19 20 - - - - - L L
< 10.0 kb
< 3.0 kb
< 1.0 kb
< 0.5 kb
L L - 21 22 23 24 25 26 27 28 29 30 - - - - - L L
< 10.0 kb
< 3.0 kb
< 1.0 kb
< 0.5 kb

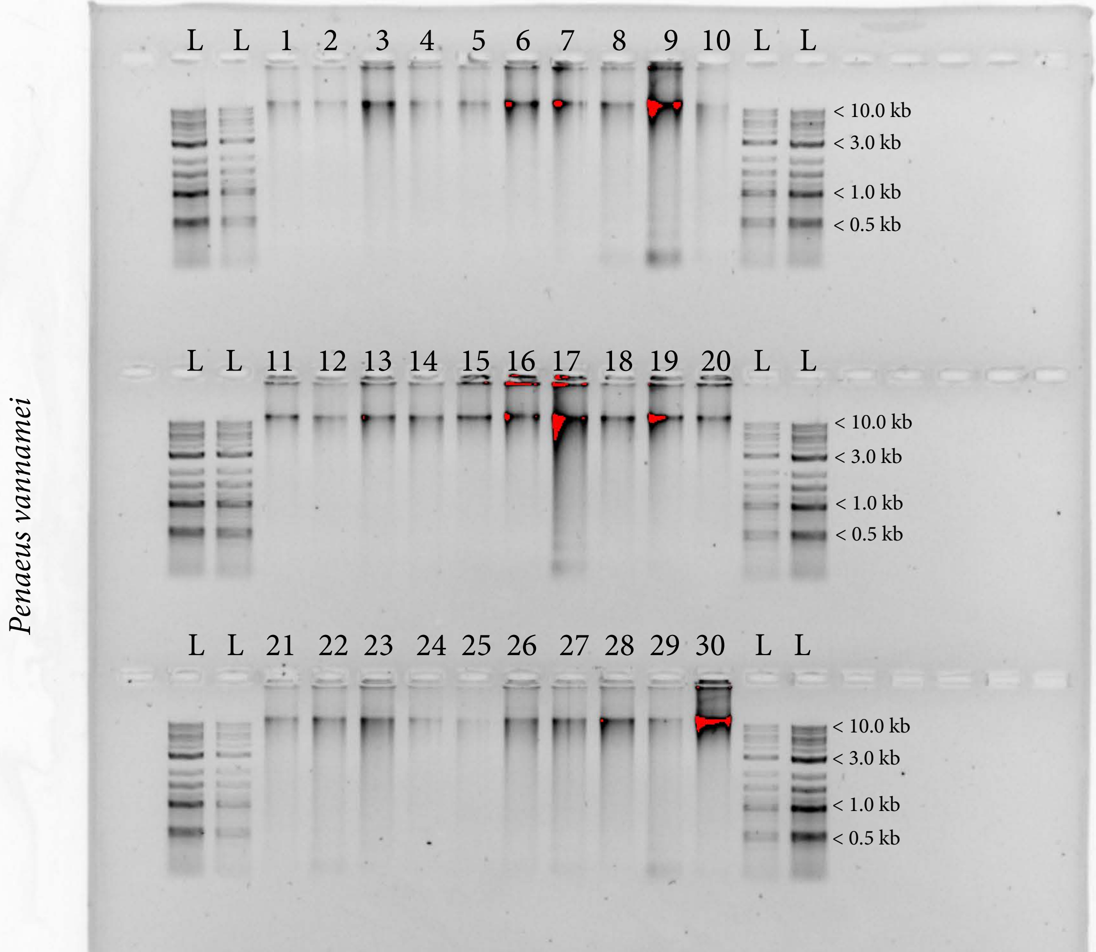
Penaeus vannamei
L L 1 2 3 4 5 6 7 8 9 10 L L
< 10.0 kb
< 3.0 kb
< 1.0 kb
< 0.5 kb
L L 11 12 13 14 15 16 17 18 19 20 L L
< 10.0 kb
< 3.0 kb
< 1.0 kb
< 0.5 kb
L L 21 22 23 24 25 26 27 28 29 30 L L
< 10.0 kb
< 3.0 kb
< 1.0 kb
< 0.5 kb
