# Supplementary figures and images for: Perish the thawed? EDTA reduces DNA degradation during extraction from frozen tissue
Source: PLoS One. 2025 Jun 3;20(6):e0321872. doi: 10.1371/journal.pone.0321872 (PMC12132941; doi:10.1371/journal.pone.0321872)

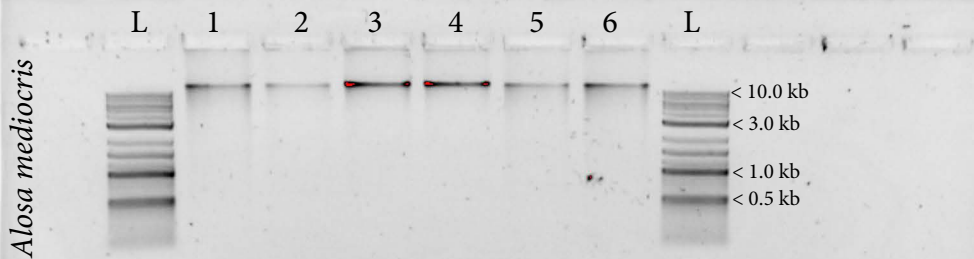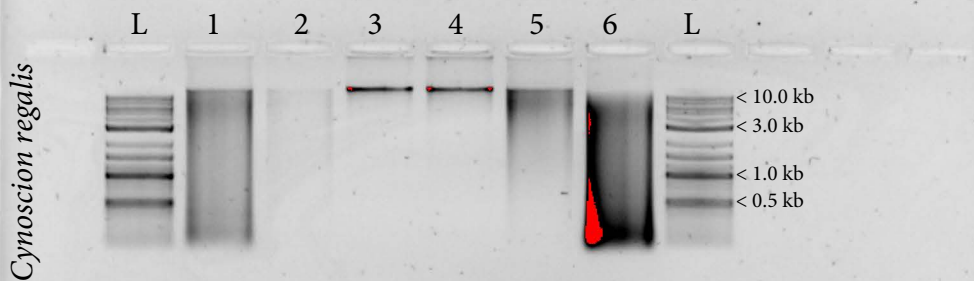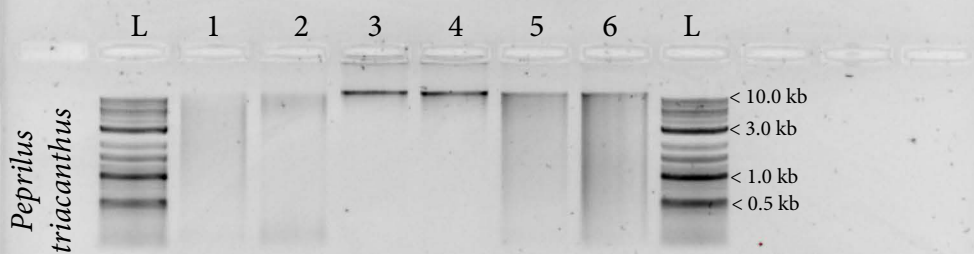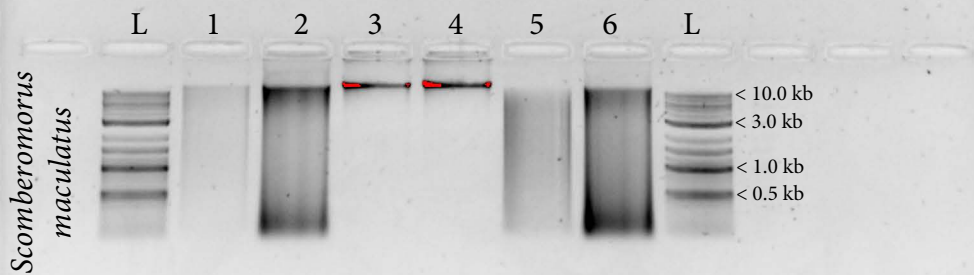

*Brevoortia tyrannus*

L 1 2 3 4 5 6 L

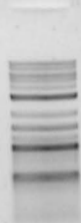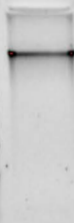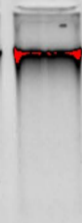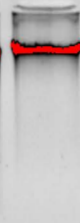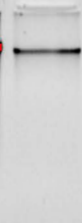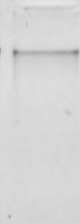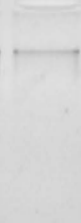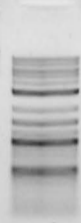

< 10.0 kb  
< 3.0 kb  
< 1.0 kb  
< 0.5 kb

*Trinectes  
maculatus*

L 1 2 3 4 5 6 L

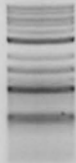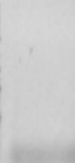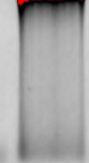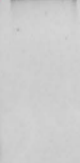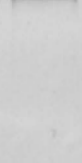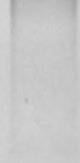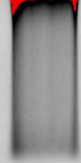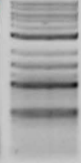

< 10.0 kb  
< 3.0 kb  
< 1.0 kb  
< 0.5 kb

Supplement: S4 Fig — DNA was extracted from tissues of six additional marine fish species that were thawed in EDTA (250 mM, pH 10; lanes 3–4) or ethanol (95%; lanes 5–6) overnight at 4°C or extracted directly from frozen tissues without subsequent liquid preservative treatment (lanes 1–2) from two randomly selected specimens of each species. Lanes marked with an L contain 0.66 μL of Quick Load Purple 1 kb Plus DNA Ladder (100 μg/mL; New England Biolabs; Ipswich, MA). Specimens are presented in the same order across all treatments. (PDF) [file pone.0321872.s004.pdf]
