# Supplementary material for: Perish the thawed? EDTA reduces DNA degradation during extraction from frozen tissue
Source: PLoS One. 2025 Jun 3;20(6):e0321872. doi: 10.1371/journal.pone.0321872 (PMC12132941; doi:10.1371/journal.pone.0321872)
Supplement: S1 Table — Taxonomic, source, collection, and storage information as well as Ocean Genome Legacy (OGL) catalog and NCBI accession numbers are presented for specimens of 16 marine fish and invertebrate species. N/a indicates samples for which NCBI accession numbers were not assigned due to sequencing failures. (PDF) [file pone.0321872.s005.pdf]

Phylum	Family	Species	Common Name	Specimen	Specimen Source	Specimen Collection Date	Specimen Storage Temperature Prior to Acquisition	Years Specimen was Frozen	OGL Catalog Number	NCBI Accession Number
Chordata	Serranidae	*Centropristis striata*	Black sea bass	1	Massachusetts Department of Marine Fisheries	12-Jul-2013	-23°C	9.8	https://arctos.database.museum/guid/OGL:Genomic:36485	PQ208355
Chordata	Serranidae	*Centropristis striata*	Black sea bass	2	Massachusetts Department of Marine Fisheries	5-Jul-2013	-23°C	9.9	https://arctos.database.museum/guid/OGL:Genomic:36486	PQ208356
Chordata	Serranidae	*Centropristis striata*	Black sea bass	3	Massachusetts Department of Marine Fisheries	13-Jul-2013	-23°C	9.8	https://arctos.database.museum/guid/OGL:Genomic:36487	PQ208357
Chordata	Serranidae	*Centropristis striata*	Black sea bass	4	Massachusetts Department of Marine Fisheries	13-Jul-2013	-23°C	9.8	https://arctos.database.museum/guid/OGL:Genomic:36488	PQ208358
Chordata	Serranidae	*Centropristis striata*	Black sea bass	5	Massachusetts Department of Marine Fisheries	13-Jul-2013	-23°C	9.8	https://arctos.database.museum/guid/OGL:Genomic:36489	PQ208359
Chordata	Serranidae	*Centropristis striata*	Black sea bass	6	Massachusetts Department of Marine Fisheries	17-Jun-2013	-23°C	9.9	https://arctos.database.museum/guid/OGL:Genomic:36490	PQ208360
Chordata	Serranidae	*Centropristis striata*	Black sea bass	7	Massachusetts Department of Marine Fisheries	17-Jun-2013	-23°C	9.9	https://arctos.database.museum/guid/OGL:Genomic:36491	PQ208361
Chordata	Serranidae	*Centropristis striata*	Black sea bass	8	Massachusetts Department of Marine Fisheries	5-Jul-2013	-23°C	9.9	https://arctos.database.museum/guid/OGL:Genomic:36492	PQ208362
Chordata	Serranidae	*Centropristis striata*	Black sea bass	9	Massachusetts Department of Marine Fisheries	19-Jun-2013	-23°C	9.9	https://arctos.database.museum/guid/OGL:Genomic:36493	PQ208363
Chordata	Serranidae	*Centropristis striata*	Black sea bass	10	Massachusetts Department of Marine Fisheries	16-Jul-2013	-23°C	9.8	https://arctos.database.museum/guid/OGL:Genomic:36494	PQ208364
Chordata	Scomberesocidae	*Cololabis saira*	Pacific saury	1	Hmart (Cambridge, MA)	unknown	unknown	unknown	https://arctos.database.museum/guid/OGL:Genomic:35467	PQ208368
Chordata	Scomberesocidae	*Cololabis saira*	Pacific saury	2	Hmart (Cambridge, MA)	unknown	unknown	unknown	https://arctos.database.museum/guid/OGL:Genomic:35468	PQ208374
Chordata	Scomberesocidae	*Cololabis saira*	Pacific saury	3	Hmart (Cambridge, MA)	unknown	unknown	unknown	https://arctos.database.museum/guid/OGL:Genomic:35469	PQ208365
Chordata	Scomberesocidae	*Cololabis saira*	Pacific saury	4	Hmart (Cambridge, MA)	unknown	unknown	unknown	https://arctos.database.museum/guid/OGL:Genomic:35470	PQ208366
Chordata	Scomberesocidae	*Cololabis saira*	Pacific saury	5	Hmart (Cambridge, MA)	unknown	unknown	unknown	https://arctos.database.museum/guid/OGL:Genomic:35471	PQ208367
Chordata	Scomberesocidae	*Cololabis saira*	Pacific saury	6	Hmart (Cambridge, MA)	unknown	unknown	unknown	https://arctos.database.museum/guid/OGL:Genomic:35472	PQ208369
Chordata	Scomberesocidae	*Cololabis saira*	Pacific saury	7	Hmart (Cambridge, MA)	unknown	unknown	unknown	https://arctos.database.museum/guid/OGL:Genomic:35473	PQ208370
Chordata	Scomberesocidae	*Cololabis saira*	Pacific saury	8	Hmart (Cambridge, MA)	unknown	unknown	unknown	https://arctos.database.museum/guid/OGL:Genomic:35474	PQ208371
Chordata	Scomberesocidae	*Cololabis saira*	Pacific saury	9	Hmart (Cambridge, MA)	unknown	unknown	unknown	https://arctos.database.museum/guid/OGL:Genomic:35475	PQ208372
Chordata	Scomberesocidae	*Cololabis saira*	Pacific saury	10	Hmart (Cambridge, MA)	unknown	unknown	unknown	https://arctos.database.museum/guid/OGL:Genomic:35476	PQ208373
Chordata	Sciaenidae	*Larimichthys polyactis*	Redlip croaker	1	Hmart (Cambridge, MA)	unknown	unknown	unknown	https://arctos.database.museum/guid/OGL:Genomic:35487	PQ208391
Chordata	Sciaenidae	*Larimichthys polyactis*	Redlip croaker	2	Hmart (Cambridge, MA)	unknown	unknown	unknown	https://arctos.database.museum/guid/OGL:Genomic:35488	PQ208397
Chordata	Sciaenidae	*Larimichthys polyactis*	Redlip croaker	3	Hmart (Cambridge, MA)	unknown	unknown	unknown	https://arctos.database.museum/guid/OGL:Genomic:35489	PQ208388
Chordata	Sciaenidae	*Larimichthys polyactis*	Redlip croaker	4	Hmart (Cambridge, MA)	unknown	unknown	unknown	https://arctos.database.museum/guid/OGL:Genomic:35490	PQ208389
Chordata	Sciaenidae	*Larimichthys polyactis*	Redlip croaker	5	Hmart (Cambridge, MA)	unknown	unknown	unknown	https://arctos.database.museum/guid/OGL:Genomic:35491	PQ208390
Chordata	Sciaenidae	*Larimichthys polyactis*	Redlip croaker	6	Hmart (Cambridge, MA)	unknown	unknown	unknown	https://arctos.database.museum/guid/OGL:Genomic:35492	PQ208392
Chordata	Sciaenidae	*Larimichthys polyactis*	Redlip croaker	7	Hmart (Cambridge, MA)	unknown	unknown	unknown	https://arctos.database.museum/guid/OGL:Genomic:35493	PQ208393
Chordata	Sciaenidae	*Larimichthys polyactis*	Redlip croaker	8	Hmart (Cambridge, MA)	unknown	unknown	unknown	https://arctos.database.museum/guid/OGL:Genomic:35494	PQ208394
Chordata	Sciaenidae	*Larimichthys polyactis*	Redlip croaker	9	Hmart (Cambridge, MA)	unknown	unknown	unknown	https://arctos.database.museum/guid/OGL:Genomic:35495	PQ208395
Chordata	Sciaenidae	*Larimichthys polyactis*	Redlip croaker	10	Hmart (Cambridge, MA)	unknown	unknown	unknown	https://arctos.database.museum/guid/OGL:Genomic:35496	PQ208396
Chordata	Atherinopsidae	*Odontesthes regia*	Silverside	1	Hmart (Cambridge, MA)	unknown	unknown	unknown	https://arctos.database.museum/guid/OGL:Genomic:35477	PQ208421
Chordata	Atherinopsidae	*Odontesthes regia*	Silverside	2	Hmart (Cambridge, MA)	unknown	unknown	unknown	https://arctos.database.museum/guid/OGL:Genomic:35478	PQ208427
Chordata	Atherinopsidae	*Odontesthes regia*	Silverside	3	Hmart (Cambridge, MA)	unknown	unknown	unknown	https://arctos.database.museum/guid/OGL:Genomic:35479	PQ208418
Chordata	Atherinopsidae	*Odontesthes regia*	Silverside	4	Hmart (Cambridge, MA)	unknown	unknown	unknown	https://arctos.database.museum/guid/OGL:Genomic:35480	PQ208419
Chordata	Atherinopsidae	*Odontesthes regia*	Silverside	5	Hmart (Cambridge, MA)	unknown	unknown	unknown	https://arctos.database.museum/guid/OGL:Genomic:35481	PQ208420
Chordata	Atherinopsidae	*Odontesthes regia*	Silverside	6	Hmart (Cambridge, MA)	unknown	unknown	unknown	https://arctos.database.museum/guid/OGL:Genomic:35482	PQ208422
Chordata	Atherinopsidae	*Odontesthes regia*	Silverside	7	Hmart (Cambridge, MA)	unknown	unknown	unknown	https://arctos.database.museum/guid/OGL:Genomic:35483	PQ208423
Chordata	Atherinopsidae	*Odontesthes regia*	Silverside	8	Hmart (Cambridge, MA)	unknown	unknown	unknown	https://arctos.database.museum/guid/OGL:Genomic:35484	PQ208424
Chordata	Atherinopsidae	*Odontesthes regia*	Silverside	9	Hmart (Cambridge, MA)	unknown	unknown	unknown	https://arctos.database.museum/guid/OGL:Genomic:35485	PQ208425
Chordata	Atherinopsidae	*Odontesthes regia*	Silverside	10	Hmart (Cambridge, MA)	unknown	unknown	unknown	https://arctos.database.museum/guid/OGL:Genomic:35486	PQ208426
Chordata	Alosidae	*Sardina pilchardus*	European pilchard	1	Market Basket (Lynn, MA)	unkown	unknown	unknown	https://arctos.database.museum/guid/OGL:Genomic:36682	PQ208444
Chordata	Alosidae	*Sardina pilchardus*	European pilchard	2	Market Basket (Lynn, MA)	unkown	unknown	unknown	https://arctos.database.museum/guid/OGL:Genomic:36683	PQ208450
Chordata	Alosidae	*Sardina pilchardus*	European pilchard	3	Market Basket (Lynn, MA)	unkown	unknown	unknown	https://arctos.database.museum/guid/OGL:Genomic:36684	PQ208441
Chordata	Alosidae	*Sardina pilchardus*	European pilchard	4	Market Basket (Lynn, MA)	unkown	unknown	unknown	https://arctos.database.museum/guid/OGL:Genomic:36685	PQ208442
Chordata	Alosidae	*Sardina pilchardus*	European pilchard	5	Market Basket (Lynn, MA)	unkown	unknown	unknown	https://arctos.database.museum/guid/OGL:Genomic:36686	PQ208443
Chordata	Alosidae	*Sardina pilchardus*	European pilchard	6	Market Basket (Lynn, MA)	unkown	unknown	unknown	https://arctos.database.museum/guid/OGL:Genomic:36687	PQ208445
Chordata	Alosidae	*Sardina pilchardus*	European pilchard	7	Market Basket (Lynn, MA)	unkown	unknown	unknown	https://arctos.database.museum/guid/OGL:Genomic:36688	PQ208446
Chordata	Alosidae	*Sardina pilchardus*	European pilchard	8	Market Basket (Lynn, MA)	unkown	unknown	unknown	https://arctos.database.museum/guid/OGL:Genomic:36689	PQ208447
Chordata	Alosidae	*Sardina pilchardus*	European pilchard	9	Market Basket (Lynn, MA)	unkown	unknown	unknown	https://arctos.database.museum/guid/OGL:Genomic:36690	PQ208448
Chordata	Alosidae	*Sardina pilchardus*	European pilchard	10	Market Basket (Lynn, MA)	unkown	unknown	unknown	https://arctos.database.museum/guid/OGL:Genomic:36691	PQ208449
Mollusca	Octopodidae	*Amphioctopus aegina*	Marbled octopus	2	Hmart (Cambridge, MA)	unknown	unknown	unknown	https://arctos.database.museum/guid/OGL:Genomic:40148	PQ208339
Mollusca	Octopodidae	*Amphioctopus aegina*	Marbled octopus	3	Hmart (Cambridge, MA)	unknown	unknown	unknown	https://arctos.database.museum/guid/OGL:Genomic:40149	PQ208340
Mollusca	Octopodidae	*Amphioctopus aegina*	Marbled octopus	4	Hmart (Cambridge, MA)	unknown	unknown	unknown	https://arctos.database.museum/guid/OGL:Genomic:40150	PQ208341
Mollusca	Octopodidae	*Amphioctopus aegina*	Marbled octopus	5	Hmart (Cambridge, MA)	unknown	unknown	unknown	https://arctos.database.museum/guid/OGL:Genomic:40151	PQ208342
Mollusca	Octopodidae	*Amphioctopus aegina*	Marbled octopus	6	Hmart (Cambridge, MA)	unknown	unknown	unknown	https://arctos.database.museum/guid/OGL:Genomic:40152	PQ208343
Mollusca	Octopodidae	*Amphioctopus aegina*	Marbled octopus	7	Hmart (Cambridge, MA)	unknown	unknown	unknown	https://arctos.database.museum/guid/OGL:Genomic:40153	PQ208344
Mollusca	Octopodidae	*Amphioctopus aegina*	Marbled octopus	9	Hmart (Cambridge, MA)	unknown	unknown	unknown	https://arctos.database.museum/guid/OGL:Genomic:40155	PQ208345
Mollusca	Octopodidae	*Amphioctopus fangsiao*	Webfoot octopus	8	Hmart (Cambridge, MA)	unknown	unknown	unknown	https://arctos.database.museum/guid/OGL:Genomic:40154	PQ208347
Mollusca	Octopodidae	*Amphioctopus fangsiao*	Webfoot octopus	10	Hmart (Cambridge, MA)	unknown	unknown	unknown	https://arctos.database.museum/guid/OGL:Genomic:40156	PQ208348
Mollusca	Octopodidae	*Amphioctopus aegina*	Marbled octopus	11	Hmart (Cambridge, MA)	unknown	unknown	unknown	https://arctos.database.museum/guid/OGL:Genomic:40157	PQ208346
Arthropoda	Nephropidae	*Homarus americanus*	American lobster	1	Ipswich Shellfish Fish Market (Ipswich, MA)	25-Feb-2020	n/a, obtained live	2.1	https://arctos.database.museum/guid/OGL:Genomic:32111	PQ208378
Arthropoda	Nephropidae	*Homarus americanus*	American lobster	2	Ipswich Shellfish Fish Market (Ipswich, MA)	25-Feb-2020	n/a, obtained live	2.1	https://arctos.database.museum/guid/OGL:Genomic:32112	PQ208379
Arthropoda	Nephropidae	*Homarus americanus*	American lobster	3	Ipswich Shellfish Fish Market (Ipswich, MA)	25-Feb-2020	n/a, obtained live	2.1	https://arctos.database.museum/guid/OGL:Genomic:32113	PQ208380
Arthropoda	Nephropidae	*Homarus americanus*	American lobster	4	Ipswich Shellfish Fish Market (Ipswich, MA)	25-Feb-2020	n/a, obtained live	2.1	https://arctos.database.museum/guid/OGL:Genomic:32114	PQ208381
Arthropoda	Nephropidae	*Homarus americanus*	American lobster	5	Ipswich Shellfish Fish Market (Ipswich, MA)	25-Feb-2020	n/a, obtained live	2.1	https://arctos.database.museum/guid/OGL:Genomic:32115	PQ208382
Arthropoda	Nephropidae	*Homarus americanus*	American lobster	6	Ipswich Shellfish Fish Market (Ipswich, MA)	25-Feb-2020	n/a, obtained live	2.1	https://arctos.database.museum/guid/OGL:Genomic:32116	PQ208383
Arthropoda	Nephropidae	*Homarus americanus*	American lobster	7	Ipswich Shellfish Fish Market (Ipswich, MA)	25-Feb-2020	n/a, obtained live	2.1	https://arctos.database.museum/guid/OGL:Genomic:32117	PQ208384
Arthropoda	Nephropidae	*Homarus americanus*	American lobster	8	Ipswich Shellfish Fish Market (Ipswich, MA)	25-Feb-2020	n/a, obtained live	2.1	https://arctos.database.museum/guid/OGL:Genomic:32118	PQ208385
Arthropoda	Nephropidae	*Homarus americanus*	American lobster	9	Ipswich Shellfish Fish Market (Ipswich, MA)	25-Feb-2020	n/a, obtained live	2.1	https://arctos.database.museum/guid/OGL:Genomic:32119	PQ208386
Arthropoda	Nephropidae	*Homarus americanus*	American lobster	10	Ipswich Shellfish Fish Market (Ipswich, MA)	25-Feb-2020	n/a, obtained live	2.1	https://arctos.database.museum/guid/OGL:Genomic:32120	PQ208387
Mollusca	Ostreidae	*Magallana gigas*	Pacific oyster	1	Hmart (Cambridge, MA)	unknown	unknown	unknown	https://arctos.database.museum/guid/OGL:Genomic:40142	PQ208401
Mollusca	Ostreidae	*Magallana gigas*	Pacific oyster	2	Hmart (Cambridge, MA)	unknown	unknown	unknown	https://arctos.database.museum/guid/OGL:Genomic:40143	PQ208407
Mollusca	Ostreidae	*Magallana gigas*	Pacific oyster	3	Hmart (Cambridge, MA)	unknown	unknown	unknown	https://arctos.database.museum/guid/OGL:Genomic:40144	PQ208398
Mollusca	Ostreidae	*Magallana gigas*	Pacific oyster	4	Hmart (Cambridge, MA)	unknown	unknown	unknown	https://arctos.database.museum/guid/OGL:Genomic:40165	PQ208399

Phylum	Family	Species	Common Name	Specimen	Specimen Source	Specimen Collection Date	Specimen Storage Temperature Prior to Acquisition	Years Specimen was Frozen	OGL Catalog Number	NCBI Accession Number
Mollusca	Ostreidae	*Magallana gigas*	Pacific oyster	5	Hmart (Cambridge, MA)	unknown	unknown	unknown	https://arctos.database.museum/guid/OGL:Genomic:40166	PQ208400
Mollusca	Ostreidae	*Magallana gigas*	Pacific oyster	6	Hmart (Cambridge, MA)	unknown	unknown	unknown	https://arctos.database.museum/guid/OGL:Genomic:40167	PQ208402
Mollusca	Ostreidae	*Magallana gigas*	Pacific oyster	7	Hmart (Cambridge, MA)	unknown	unknown	unknown	https://arctos.database.museum/guid/OGL:Genomic:40168	PQ208403
Mollusca	Ostreidae	*Magallana gigas*	Pacific oyster	8	Hmart (Cambridge, MA)	unknown	unknown	unknown	https://arctos.database.museum/guid/OGL:Genomic:40169	PQ208404
Mollusca	Ostreidae	*Magallana gigas*	Pacific oyster	9	Hmart (Cambridge, MA)	unknown	unknown	unknown	https://arctos.database.museum/guid/OGL:Genomic:40170	PQ208405
Mollusca	Ostreidae	*Magallana gigas*	Pacific oyster	10	Hmart (Cambridge, MA)	unknown	unknown	unknown	https://arctos.database.museum/guid/OGL:Genomic:40171	PQ208406
Mollusca	Veneridae	*Mercenaria mercenaria*	Hard-shell clam	1	Cape Cod Shellfish & Seafood Co. (Boston, MA)	2-Mar-2020	n/a, obtained live	2.1	https://arctos.database.museum/guid/OGL:Genomic:32121	PQ208408
Mollusca	Veneridae	*Mercenaria mercenaria*	Hard-shell clam	2	Cape Cod Shellfish & Seafood Co. (Boston, MA)	2-Mar-2020	n/a, obtained live	2.1	https://arctos.database.museum/guid/OGL:Genomic:32122	PQ208409
Mollusca	Veneridae	*Mercenaria mercenaria*	Hard-shell clam	3	Cape Cod Shellfish & Seafood Co. (Boston, MA)	2-Mar-2020	n/a, obtained live	2.1	https://arctos.database.museum/guid/OGL:Genomic:32123	PQ208410
Mollusca	Veneridae	*Mercenaria mercenaria*	Hard-shell clam	4	Cape Cod Shellfish & Seafood Co. (Boston, MA)	2-Mar-2020	n/a, obtained live	2.1	https://arctos.database.museum/guid/OGL:Genomic:32124	PQ208411
Mollusca	Veneridae	*Mercenaria mercenaria*	Hard-shell clam	5	Cape Cod Shellfish & Seafood Co. (Boston, MA)	2-Mar-2020	n/a, obtained live	2.1	https://arctos.database.museum/guid/OGL:Genomic:32125	PQ208412
Mollusca	Veneridae	*Mercenaria mercenaria*	Hard-shell clam	6	Cape Cod Shellfish & Seafood Co. (Boston, MA)	2-Mar-2020	n/a, obtained live	2.1	https://arctos.database.museum/guid/OGL:Genomic:32126	PQ208413
Mollusca	Veneridae	*Mercenaria mercenaria*	Hard-shell clam	7	Cape Cod Shellfish & Seafood Co. (Boston, MA)	2-Mar-2020	n/a, obtained live	2.1	https://arctos.database.museum/guid/OGL:Genomic:32127	PQ208414
Mollusca	Veneridae	*Mercenaria mercenaria*	Hard-shell clam	8	Cape Cod Shellfish & Seafood Co. (Boston, MA)	2-Mar-2020	n/a, obtained live	2.1	https://arctos.database.museum/guid/OGL:Genomic:32128	PQ208415
Mollusca	Veneridae	*Mercenaria mercenaria*	Hard-shell clam	9	Cape Cod Shellfish & Seafood Co. (Boston, MA)	2-Mar-2020	n/a, obtained live	2.1	https://arctos.database.museum/guid/OGL:Genomic:32129	PQ208416
Mollusca	Veneridae	*Mercenaria mercenaria*	Hard-shell clam	10	Cape Cod Shellfish & Seafood Co. (Boston, MA)	2-Mar-2020	n/a, obtained live	2.1	https://arctos.database.museum/guid/OGL:Genomic:32130	PQ208417
Arthropoda	Penaeidae	*Penaeus vannamei*	Whiteleg shrimp	1	Hmart (Cambridge, MA)	unknown	unknown	unknown	https://arctos.database.museum/guid/OGL:Genomic:40158	PQ208431
Arthropoda	Penaeidae	*Penaeus vannamei*	Whiteleg shrimp	2	Hmart (Cambridge, MA)	unknown	unknown	unknown	https://arctos.database.museum/guid/OGL:Genomic:40159	PQ208437
Arthropoda	Penaeidae	*Penaeus vannamei*	Whiteleg shrimp	3	Hmart (Cambridge, MA)	unknown	unknown	unknown	https://arctos.database.museum/guid/OGL:Genomic:40160	PQ208428
Arthropoda	Penaeidae	*Penaeus vannamei*	Whiteleg shrimp	4	Hmart (Cambridge, MA)	unknown	unknown	unknown	https://arctos.database.museum/guid/OGL:Genomic:40161	PQ208429
Arthropoda	Penaeidae	*Penaeus vannamei*	Whiteleg shrimp	5	Hmart (Cambridge, MA)	unknown	unknown	unknown	https://arctos.database.museum/guid/OGL:Genomic:40162	PQ208430
Arthropoda	Penaeidae	*Penaeus vannamei*	Whiteleg shrimp	6	Hmart (Cambridge, MA)	unknown	unknown	unknown	https://arctos.database.museum/guid/OGL:Genomic:40163	PQ208432
Arthropoda	Penaeidae	*Penaeus vannamei*	Whiteleg shrimp	7	Hmart (Cambridge, MA)	unknown	unknown	unknown	https://arctos.database.museum/guid/OGL:Genomic:40164	PQ208433
Arthropoda	Penaeidae	*Penaeus vannamei*	Whiteleg shrimp	8	Hmart (Cambridge, MA)	unknown	unknown	unknown	https://arctos.database.museum/guid/OGL:Genomic:40145	PQ208434
Arthropoda	Penaeidae	*Penaeus vannamei*	Whiteleg shrimp	9	Hmart (Cambridge, MA)	unknown	unknown	unknown	https://arctos.database.museum/guid/OGL:Genomic:40146	PQ208435
Arthropoda	Penaeidae	*Penaeus vannamei*	Whiteleg shrimp	10	Hmart (Cambridge, MA)	unknown	unknown	unknown	https://arctos.database.museum/guid/OGL:Genomic:40141	PQ208436
Chordata	Alosidae	*Alosa mediocris*	Hickory shad	1	Connecticut Department of Energy and Environmental Protection	5-Sep-2019	-80°C	2.9	https://arctos.database.museum/guid/OGL:Genomic:30366	PQ208336
Chordata	Alosidae	*Alosa mediocris*	Hickory shad	2	Connecticut Department of Energy and Environmental Protection	5-Sep-2019	-80°C	2.9	https://arctos.database.museum/guid/OGL:Genomic:30367	PQ208337
Chordata	Alosidae	*Alosa mediocris*	Hickory shad	3	Connecticut Department of Energy and Environmental Protection	21-Oct-2019	-80°C	2.8	https://arctos.database.museum/guid/OGL:Genomic:30403	PQ208338
Chordata	Alosidae	*Brevoortia tyrannus*	Atlantic menhaden	1	Connecticut Department of Energy and Environmental Protection	5-Sep-2019	-80°C	3.1	https://arctos.database.museum/guid/OGL:Genomic:30368	PQ208352
Chordata	Alosidae	*Brevoortia tyrannus*	Atlantic menhaden	2	Connecticut Department of Energy and Environmental Protection	5-Sep-2019	-80°C	3.1	https://arctos.database.museum/guid/OGL:Genomic:30369	PQ208354
Chordata	Alosidae	*Brevoortia tyrannus*	Atlantic menhaden	3	Connecticut Department of Energy and Environmental Protection	5-Sep-2019	-80°C	3.1	https://arctos.database.museum/guid/OGL:Genomic:30370	PQ208349
Chordata	Alosidae	*Brevoortia tyrannus*	Atlantic menhaden	4	Connecticut Department of Energy and Environmental Protection	5-Sep-2019	-80°C	3.1	https://arctos.database.museum/guid/OGL:Genomic:30371	PQ208350
Chordata	Alosidae	*Brevoortia tyrannus*	Atlantic menhaden	5	Connecticut Department of Energy and Environmental Protection	5-Sep-2019	-80°C	3.1	https://arctos.database.museum/guid/OGL:Genomic:30372	PQ208351
Chordata	Alosidae	*Brevoortia tyrannus*	Atlantic menhaden	6	Connecticut Department of Energy and Environmental Protection	5-Sep-2019	-80°C	3.1	https://arctos.database.museum/guid/OGL:Genomic:30373	PQ208353
Chordata	Sciaenidae	*Cynoscion regalis*	Weakfish	1	Connecticut Department of Energy and Environmental Protection	5-Sep-2019	-80°C	2.9	https://arctos.database.museum/guid/OGL:Genomic:30391	PQ208376
Chordata	Sciaenidae	*Cynoscion regalis*	Weakfish	2	Connecticut Department of Energy and Environmental Protection	5-Sep-2019	-80°C	2.9	https://arctos.database.museum/guid/OGL:Genomic:30392	PQ208377
Chordata	Sciaenidae	*Cynoscion regalis*	Weakfish	3	Connecticut Department of Energy and Environmental Protection	5-Sep-2019	-80°C	2.9	https://arctos.database.museum/guid/OGL:Genomic:30393	PQ208375
Chordata	Stromateidae	*Peprilus triacanthus*	Atlantic butterfish	1	Connecticut Department of Energy and Environmental Protection	25-Oct-2018	-80°C	3.8	https://arctos.database.museum/guid/OGL:Genomic:30337	PQ208439
Chordata	Stromateidae	*Peprilus triacanthus*	Atlantic butterfish	2	Connecticut Department of Energy and Environmental Protection	25-Oct-2018	-80°C	3.8	https://arctos.database.museum/guid/OGL:Genomic:30338	PQ208440
Chordata	Stromateidae	*Peprilus triacanthus*	Atlantic butterfish	3	Connecticut Department of Energy and Environmental Protection	25-Oct-2018	-80°C	3.8	https://arctos.database.museum/guid/OGL:Genomic:30339	PQ208438
Chordata	Scombridae	*Scomberomorus maculatus*	Spanish mackerel	1	Connecticut Department of Energy and Environmental Protection	5-Sep-2019	-80°C	2.9	https://arctos.database.museum/guid/OGL:Genomic:30384	PQ208454
Chordata	Scombridae	*Scomberomorus maculatus*	Spanish mackerel	2	Connecticut Department of Energy and Environmental Protection	5-Sep-2019	-80°C	2.9	https://arctos.database.museum/guid/OGL:Genomic:30385	PQ208456
Chordata	Scombridae	*Scomberomorus maculatus*	Spanish mackerel	3	Connecticut Department of Energy and Environmental Protection	5-Sep-2019	-80°C	2.9	https://arctos.database.museum/guid/OGL:Genomic:30386	PQ208451
Chordata	Scombridae	*Scomberomorus maculatus*	Spanish mackerel	4	Connecticut Department of Energy and Environmental Protection	5-Sep-2019	-80°C	3.1	https://arctos.database.museum/guid/OGL:Genomic:30387	PQ208452
Chordata	Scombridae	*Scomberomorus maculatus*	Spanish mackerel	5	Connecticut Department of Energy and Environmental Protection	5-Sep-2019	-80°C	3.1	https://arctos.database.museum/guid/OGL:Genomic:30388	PQ208453
Chordata	Scombridae	*Scomberomorus maculatus*	Spanish mackerel	6	Connecticut Department of Energy and Environmental Protection	5-Sep-2019	-80°C	3.1	https://arctos.database.museum/guid/OGL:Genomic:30389	PQ208455
Chordata	Scombridae	*Trinectes maculatus*	Hogchoker	1	Connecticut Department of Energy and Environmental Protection	30-Oct-2018	-80°C	3.8	https://arctos.database.museum/guid/OGL:Genomic:30358	n/a
Chordata	Scombridae	*Trinectes maculatus*	Hogchoker	2	Connecticut Department of Energy and Environmental Protection	30-Oct-2018	-80°C	3.8	https://arctos.database.museum/guid/OGL:Genomic:30359	n/a
Chordata	Scombridae	*Trinectes maculatus*	Hogchoker	3	Connecticut Department of Energy and Environmental Protection	5-Sep-2019	-80°C	2.9	https://arctos.database.museum/guid/OGL:Genomic:30377	n/a
Chordata	Scombridae	*Trinectes maculatus*	Hogchoker	4	Connecticut Department of Energy and Environmental Protection	5-Sep-2019	-80°C	3.1	https://arctos.database.museum/guid/OGL:Genomic:30378	n/a
