# Supplementary material for: Perish the thawed? EDTA reduces DNA degradation during extraction from frozen tissue
Source: PLoS One. 2025 Jun 3;20(6):e0321872. doi: 10.1371/journal.pone.0321872 (PMC12132941; doi:10.1371/journal.pone.0321872)
Supplement: S2 Table — Supporting data are presented for DNA extracts of tissues from specimens of 16 marine fish and invertebrate species that were thawed in EDTA (250 mM, pH 10) or ethanol (95%) overnight at 4°C or extracted directly from frozen tissues without subsequent liquid preservative treatment. Specifically, species, sample ID, specimen and replicate numbers, treatments, initial and post-treatment sample weights, correction ratios, measured and corrected weights of tissue subsamples used for DNA extraction, A260, A260/A280 ratios, and A260/A230 ratios calculated using the Nanodrop 1000 droplet spectrophotometer, total and low molecular weight (150 bp–10kb) DNA concentration values determined by the Agilent Tapestation DNA Analyzer 2200, percentages of low molecular weight (%LMW) and high molecular weight (%HMW) DNA and nY calculated based on Tapestation data, as well as mitochondrial COI PCR amplification and sequencing success are presented for each DNA sample analyzed in this study. N/a indicates samples for which data were not collected. (PDF) [file pone.0321872.s006.pdf]

Species	Sample ID	Specimen	Replicate	Treatment	Initial Sample Weight (mg)	Post-treatment Sample Weight (mg)	Correction Ratio	Weight of Tissue Used for Extraction (mg)	Corrected Weight of Tissue Used for Extraction (mg)	A_{260}	A_{260}/A_{280} Ratio	A_{260}/A_{230} Ratio	Total DNA Concentration (ng/µL)	LMW DNA Concentration (ng/µL)	%LMW	%HMW	nY (µg DNA/mg tissue)	*COI* PCR Amplification Success	*COI* Sequencing Success
Centropristis striata	MJCS11	1	1	250 mM EDTA pH 10.0	123.6	n/a	1	28.2	28.2	0.599	1.91	1.03	24.2	9.7163	40.15	59.85	0.051360638	yes	yes
Centropristis striata	MJCS12	2	1	250 mM EDTA pH 10.0	101	n/a	1	22.9	22.9	0.469	2.07	1.1	24.4	12.9442	53.05	46.95	0.050025328	yes	yes
Centropristis striata	MJCS13	3	1	250 mM EDTA pH 10.0	107.4	n/a	1	25.1	25.1	0.364	1.86	0.7	12.8	5.04064	39.38	60.62	0.030913785	yes	yes
Centropristis striata	MJCS14	4	1	250 mM EDTA pH 10.0	124.6	n/a	1	25.3	25.3	0.735	1.92	1.67	40.7	21.6931	53.3	46.7	0.075126087	yes	yes
Centropristis striata	MJCS15	5	1	250 mM EDTA pH 10.0	116.2	n/a	1	25.6	25.6	0.287	1.83	0.63	12.4	7.63096	61.54	38.46	0.018629063	yes	yes
Centropristis striata	MJCS16	6	1	250 mM EDTA pH 10.0	127.5	n/a	1	24.8	24.8	0.208	2.2	0.96	13.8	9.88908	71.66	28.34	0.015769839	yes	yes
Centropristis striata	MJCS17	7	1	250 mM EDTA pH 10.0	103.7	n/a	1	25.3	25.3	0.382	1.91	1.31	24.3	8.90352	36.64	63.36	0.060855652	yes	yes
Centropristis striata	MJCS18	8	1	250 mM EDTA pH 10.0	108	n/a	1	27.3	27.3	0.251	2.09	1.49	11.6	3.53104	30.44	69.56	0.02955663	yes	yes
Centropristis striata	MJCS19	9	1	250 mM EDTA pH 10.0	127.9	n/a	1	26.6	26.6	0.536	1.95	1.59	21	6.993	33.3	66.7	0.052657895	yes	yes
Centropristis striata	MJCS20	10	1	250 mM EDTA pH 10.0	126.5	n/a	1	20.3	20.3	0.204	1.85	0.49	7.19	2.373419	33.01	66.99	0.023727	yes	yes
Centropristis striata	MJCS31	1	2	95% EtOH	98.6	n/a	1	21.5	21.5	0.333	2.54	1.36	16.1	15.36745	95.45	4.55	0.003407209	yes	yes
Centropristis striata	MJCS32	2	2	95% EtOH	104.8	n/a	1	25.9	25.9	0.53	2.18	1.73	32.3	30.94663	95.81	4.19	0.005225367	yes	yes
Centropristis striata	MJCS33	3	2	95% EtOH	128	n/a	1	29.4	29.4	0.558	1.95	1.29	31	27.528	88.8	11.2	0.011809524	yes	yes
Centropristis striata	MJCS34	4	2	95% EtOH	98.9	n/a	1	25.4	25.4	0.542	1.91	1.28	28.3	24.94645	88.15	11.85	0.013202953	yes	yes
Centropristis striata	MJCS35	5	2	95% EtOH	115	n/a	1	28.2	28.2	1.404	1.96	2.03	74.4	69.28872	93.13	6.87	0.018125106	yes	yes
Centropristis striata	MJCS36	6	2	95% EtOH	107.7	n/a	1	20.7	20.7	0.279	1.94	1.56	11.3	10.42877	92.29	7.71	0.004208841	yes	yes
Centropristis striata	MJCS37	7	2	95% EtOH	134.2	n/a	1	27.6	27.6	0.976	1.86	1.17	38.7	35.31375	91.25	8.75	0.012269022	yes	yes
Centropristis striata	MJCS38	8	2	95% EtOH	124.4	n/a	1	25.8	25.8	0.382	2.02	0.64	25.7	22.56717	87.81	12.19	0.012142752	yes	yes
Centropristis striata	MJCS39	9	2	95% EtOH	130.1	n/a	1	22.3	22.3	0.923	1.86	1.2	38.3	35.31643	92.21	7.79	0.013379238	yes	yes
Centropristis striata	MJCS40	10	2	95% EtOH	113.7	n/a	1	24.4	24.4	0.449	1.9	0.8	22.3	17.01267	76.29	23.71	0.021669385	yes	yes
Centropristis striata	MJCS01	1	3	Frozen, No preservative	111.7	n/a	1	23.3	23.3	0.334	1.95	0.79	21.1	19.99647	94.77	5.23	0.00473618	yes	yes
Centropristis striata	MJCS02	2	3	Frozen, No preservative	118.1	n/a	1	24.2	24.2	0.314	1.89	0.69	19	17.5636	92.44	7.56	0.005935537	yes	yes
Centropristis striata	MJCS03	3	3	Frozen, No preservative	99.8	n/a	1	24	24	0.39	1.82	0.77	23.1	20.2125	87.5	12.5	0.01203125	yes	yes
Centropristis striata	MJCS04	4	3	Frozen, No preservative	111.4	n/a	1	30.6	30.6	0.276	2.01	0.84	14.8	12.57556	84.97	15.03	0.007269412	yes	yes
Centropristis striata	MJCS05	5	3	Frozen, No preservative	105.6	n/a	1	26.1	26.1	0.321	1.77	0.6	13.5	11.86515	87.89	12.11	0.006263793	yes	yes
Centropristis striata	MJCS06	6	3	Frozen, No preservative	111.3	n/a	1	28.4	28.4	0.255	1.79	0.25	4.23	3.656835	86.45	13.55	0.002018187	no	no
Centropristis striata	MJCS07	7	3	Frozen, No preservative	119	n/a	1	28.5	28.5	0.637	1.82	1	30.3	21.49179	70.93	29.07	0.030906	yes	yes
Centropristis striata	MJCS08	8	3	Frozen, No preservative	120.5	n/a	1	24.8	24.8	0.577	1.87	1.17	26.6	24.20068	90.98	9.02	0.009674677	yes	yes
Centropristis striata	MJCS09	9	3	Frozen, No preservative	121.5	n/a	1	27.1	27.1	0.826	1.83	1.27	36.5	28.47365	78.01	21.99	0.029617528	yes	yes
Centropristis striata	MJCS10	10	3	Frozen, No preservative	122.6	n/a	1	25.2	25.2	0.291	1.93	0.85	15.7	12.06859	76.87	23.13	0.014410357	yes	yes
Cololabis saira	EMMA2	1	1	250 mM EDTA pH 10.0	104.3	120.3	0.866999169	22.4	19.42078138	1.641	1.89	1.51	123	60.0363	48.81	51.19	0.324207862	yes	yes
Cololabis saira	EMMA6	2	1	250 mM EDTA pH 10.0	98.7	159.5	0.618808777	26.2	16.21278997	0.37	3.12	1.05	37.8	5.9724	15.8	84.2	0.196311678	yes	yes
Cololabis saira	EMMA10	3	1	250 mM EDTA pH 10.0	107.2	141.4	0.758132956	25.9	19.63564356	1.374	1.89	1.4	90.6	41.77566	46.11	53.89	0.24865159	yes	yes
Cololabis saira	EMMA14	4	1	250 mM EDTA pH 10.0	104.1	141.7	0.73465067	18.4	13.51757234	2.168	1.7	1.06	64.5	49.7037	77.06	22.94	0.109459743	yes	yes
Cololabis saira	EMMA18	5	1	250 mM EDTA pH 10.0	106.6	147.1	0.72467709	22.7	16.45016995	0.641	1.96	0.88	27.6	3.80604	13.79	86.21	0.144642639	yes	yes
Cololabis saira	EMMA22	6	1	250 mM EDTA pH 10.0	109.1	146.6	0.74420191	19.8	14.73519782	1.623	1.92	1.78	89	52.7325	59.25	40.75	0.246128355	yes	yes
Cololabis saira	EMMA26	7	1	250 mM EDTA pH 10.0	98.5	126.5	0.778656126	22.2	17.28616601	1.958	1.91	1.55	103	26.3062	25.54	74.46	0.443671546	yes	yes
Cololabis saira	EMMA30	8	1	250 mM EDTA pH 10.0	97.4	130.4	0.746932515	23.7	17.70230061	1.152	1.93	1.86	64.1	20.99916	32.76	67.24	0.243475924	yes	yes
Cololabis saira	EMMA34	9	1	250 mM EDTA pH 10.0	95.2	121.3	0.784830998	29	22.76009893	1.987	1.73	0.74	94.3	34.78727	36.89	63.11	0.261478345	yes	yes
Cololabis saira	EMMA38	10	1	250 mM EDTA pH 10.0	89.7	110.7	0.810298103	23.3	18.8799458	3.393	1.63	0.84	87.2	26.9012	30.85	69.15	0.319380154	yes	yes
Cololabis saira	EMMA3	1	2	95% EtOH	109	93.8	1.162046908	23.6	27.42430704	4.588	1.71	1.27	135	95.6205	70.83	29.17	0.143593419	yes	yes
Cololabis saira	EMMA7	2	2	95% EtOH	103.3	65.9	1.567526555	21.3	33.38831563	4.602	1.79	1.49	240	140.232	58.43	41.57	0.29881112	yes	yes
Cololabis saira	EMMA11	3	2	95% EtOH	98.3	90.4	1.087389381	26.5	28.81581858	3.401	1.7	1.17	135	101.8845	75.47	24.53	0.114921254	yes	yes
Cololabis saira	EMMA15	4	2	95% EtOH	104.6	105.2	0.994296578	26.6	26.44828897	3.296	1.82	1.4	196	160.0928	81.68	18.32	0.135763792	yes	yes
Cololabis saira	EMMA19	5	2	95% EtOH	92.4	77.9	1.186136072	29.2	34.6351733	2.499	1.8	1.17	148	94.498	63.85	36.15	0.154473025	yes	yes
Cololabis saira	EMMA23	6	2	95% EtOH	92	78.5	1.171974522	28.8	33.75286624	2.411	1.85	1.66	172	117.4244	68.27	31.73	0.16169175	yes	yes
Cololabis saira	EMMA27	7	2	95% EtOH	108.1	87.1	1.241102181	26.4	32.76509759	2.138	1.87	1.8	107	87.1515	81.45	18.55	0.060578181	yes	yes
Cololabis saira	EMMA31	8	2	95% EtOH	91.5	80.3	1.139476961	24.9	28.37297634	2.601	1.83	1.24	143	105.7914	73.98	26.02	0.131140983	yes	yes
Cololabis saira	EMMA35	9	2	95% EtOH	110.7	96.9	1.142414861	25.8	29.47430341	3.675	1.73	1.19	155	97.309	62.78	37.22	0.195733209	yes	yes
Cololabis saira	EMMA39	10	2	95% EtOH	90.6	72.4	1.251381215	27.1	33.91243094	3.337	1.63	0.72	113	86.4224	76.48	23.52	0.078371262	yes	yes
Cololabis saira	EMMA1	1	3	Frozen, No preservative	95.9	93.4	1.026766595	21.4	21.97280514	1.616	1.9	2.06	114	74.9892	65.78	34.22	0.177541282	yes	yes
Cololabis saira	EMMA5	2	3	Frozen, No preservative	110.3	109	1.011926606	22	22.26238532	2.372	1.88	1.95	124	96.0008	77.42	22.58	0.125769093	yes	yes
Cololabis saira	EMMA9	3	3	Frozen, No preservative	100.3	100.3	1	27.3	27.3	1.898	1.85	2.26	112	95.8832	85.61	14.39	0.059035897	yes	yes
Cololabis saira	EMMA13	4	3	Frozen, No preservative	105.5	103.9	1.015399423	24.9	25.28344562	2.385	1.85	1.53	207	161.8533	78.19	21.81	0.178562292	yes	yes
Cololabis saira	EMMA17	5	3	Frozen, No preservative	106	97	1.092783505	23.4	25.57113402	1.679	1.77	1.09	106	69.3664	65.44	34.56	0.143261538	yes	yes
Cololabis saira	EMMA21	6	3	Frozen, No preservative	109.7	106.1	1.033930254	26.8	27.70933082	1.171	1.9	2.43	86.9	76.52414	88.06	11.94	0.037445365	yes	yes
Cololabis saira	EMMA25	7	3	Frozen, No preservative	95.6	95.8	0.997912317	27.7	27.64217119	2.459	1.9	2.23	172	136.2412	79.21	20.79	0.12936321	yes	yes
Cololabis saira	EMMA29	8	3	Frozen, No preservative	108.5	109.5	0.99086758	28.2	27.94246575	1.922	1.87	2.02	123	100.2327	81.49	18.51	0.081479209	yes	yes
Cololabis saira	EMMA33	9	3	Frozen, No preservative	99.1	97.1	1.020597322	27.2	27.76024717	2.627	1.79	0.97	115	86.94	75.6	24.4	0.101079792	yes	yes
Cololabis saira	EMMA37	10	3	Frozen, No preservative	102.3	100.1	1.021978022	29.1	29.73956044	1.7	1.89	1.53	89.4	72.38718	80.97	19.03	0.057206024	yes	yes
Larimichthys polyactis	EMLP2	1	1	250 mM EDTA pH 10.0	94.9	163.1	0.581851625	26.8	15.59362354	0.139	1.97	0.64	7.56	3.138912	41.52	58.48	0.028351896	yes	yes
Larimichthys polyactis	EMLP6	2	1	250 mM EDTA pH 10.0	105.3	162.7	0.647203442	25.8	16.6978488	0.272	1.76	1.11	11.7	4.20264	35.92	64.08	0.044900155	yes	yes
Larimichthys polyactis	EMLP10	3	1	250 mM EDTA pH 10.0	94.2	157.1	0.599618078	25.4	15.23029917	1.205	1.54	0.64	13.9	5.14022	36.98	63.02	0.057515482	yes	yes
Larimichthys polyactis	EMLP14	4	1	250 mM EDTA pH 10.0	90.7	149.2	0.607908847	25.7	15.62325737	1.837	1.5	0.68	9.07	3.833889	42.27	57.73	0.033514848	yes	yes
Larimichthys polyactis	EMLP18	5	1	250 mM EDTA pH 10.0	102.1	161.8	0.631025958	27.6	17.41631644	1.06	1.51	0.64	10.2	8.87604	87.02	12.98	0.007601837	yes	yes
Larimichthys polyactis	EMLP22	6	1	250 mM EDTA pH 10.0	95.3	145	0.657241379	26.1	17.154	0.298	1.89	0.57	12.1	4.43586	36.66	63.34	0.044678442	yes	yes
Larimichthys polyactis	EMLP26	7	1	250 mM EDTA pH 10.0	101.5	183.4	0.553435115	27.3	15.10877863	0.579	1.62	0.65	9.21	3.033774	32.94	67.06	0.040878394	yes	yes
Larimichthys polyactis	EMLP30	8	1	250 mM EDTA pH 10.0	109.5	200.4	0.546407186	24.4	13.33233533	0.177	2.33	0.45	9.5	4.37285	46.03	53.97	0.038456503	yes	yes
Larimichthys polyactis	EMLP34	9	1	250 mM EDTA pH 10.0	100.7	164.9	0.610673135	30.2	18.44232868	0.237	1.93	1.2	21.5	10.18025	47.35	52.65	0.061379179	yes	no
Larimichthys polyactis	EMLP38	10	1	250 mM EDTA pH 10.0	95.8	156.5	0.612140575	28.9	17.69086262	1.458	1.55	0.64	11.6	2.77472	23.92	76.08	0.049886092	yes	yes
Larimichthys polyactis	EMLP3	1	2	95% EtOH	98.1	90.3	1.086378738	24.5	26.61627907	1.002	1.67	0.88	28.9	19.75893	68.37	31.63	0.034343907	yes	yes
Larimichthys polyactis	EMLP7	2	2	95% EtOH	90.4	186.5	0.484718499	18	8.724932976	0.229	1.83	0.65	14.8	11.64908	78.71	21.29	0.036113974	yes	yes
Larimichthys polyactis	EMLP11	3	2	95% EtOH	107.1	84.9	1.261484099	20.4	25.73427562	0.394	1.59	0.79	20.3	9.7846	48.2	51.8	0.040861457	yes	yes
Larimichthys polyactis	EMLP15	4	2	95% EtOH	109.1	92.5	1.179459459	29.3	34.55816216	0.184	2.05	0.57	12.1	9.24803	76.43	23.57	0.008252667	yes	yes
Larimichthys polyactis	EMLP19	5	2	95% EtOH	100.6	78.9	1.275031686	21.5	27.41318124	1.354	1.54	0.7	18.9	17.78112	94.08	5.92	0.00408154	yes	yes
Larimichthys polyactis	EMLP23	6	2	95% EtOH	96.6	89	1.085393258	21.5	23.33595506	1.044	1.74	0.94	38.1	16.77162	44.02	55.98	0.091397074	yes	yes
Larimichthys polyactis	EMLP27	7	2	95% EtOH	94.5	79.3	1.191677175	22.6	26.93190416	0.393	2.11	1.02	24.8	18.63224	75.13	24.87	0.022901314	yes	yes
Larimichthys polyactis	EMLP31	8	2	95% EtOH	98.2	74	1.327027027	21.6	28.66378378	1.774	1.54	0.68	19.3	15.18524	78.68	21.32	0.014355258	yes	yes
Larimichthys polyactis	EMLP35	9	2	95% EtOH	109.6	91.3	1.200438116	27.7	33.25213582	0.465	2.13	1.15	42.3	24.33942	57.54	42.46	0.054013312	yes	yes
Larimichthys polyactis	EMLP39	10	2	95% EtOH	107.2	94.1	1.139213603	20	22.78427205	0.632	1.92	1.12	44.3	35.48873	80.11	19.89	0.038672598	yes	no
Larimichthys polyactis	EMLP1	1	3	Frozen, No preservative	102.8	96.3	1.067497404	19.5	20.81619938	0.236	2.42	0.79	16.4	10.05484	61.31	38.69	0.030481837	yes	yes
Larimichthys polyactis	EMLP5	2	3	Frozen, No preservative	96.3	95.4	1.009433962	27.8	28.06226415	1.595	1.95	2.26	88	73.7352	83.79	16.21	0.050832677	yes	yes
Larimichthys polyactis	EMLP9	3	3	Frozen, No preservative	93.6	92.4	1.012987013	20.3	20.56363636	0.169	1.81	0.75	5.69	4.107611	72.19	27.81	0.007695084	yes	yes

Species	Sample ID	Specimen	Replicate	Treatment	Initial Sample Weight (mg)	Post-treatment Sample Weight (mg)	Correction Ratio	Weight of Tissue Used for Extraction (mg)	Corrected Weight of Tissue Used for Extraction (mg)	A_{260}	A_{260}/A_{280} Ratio	A_{260}/A_{230} Ratio	Total DNA Concentration (ng/μL)	LMW DNA Concentration (ng/μL)	%LMW	%HMW	nY (μg DNA/mg tissue)	*COI* PCR Amplification Success	*COI* Sequencing Success
Larimichthys polyactis	EMLP13	4	3	Frozen, No preservative	100.6	99.5	1.011055276	21.8	22.04100503	0.043	1.17	0.67	12.3	7.90275	64.25	35.75	0.019950315	yes	yes
Larimichthys polyactis	EMLP17	5	3	Frozen, No preservative	100.3	100.5	0.99800995	25.5	25.44925373	0.825	1.81	1.29	43	39.9513	92.91	7.09	0.011979526	yes	yes
Larimichthys polyactis	EMLP21	6	3	Frozen, No preservative	93.3	92.1	1.013029316	28.5	28.8713355	0.64	1.85	0.81	41.6	24.92256	59.91	40.09	0.057764699	yes	yes
Larimichthys polyactis	EMLP25	7	3	Frozen, No preservative	106.1	102.5	1.035121951	23.8	24.63590244	2.885	1.74	1.41	126	92.5344	73.44	26.56	0.135840772	yes	yes
Larimichthys polyactis	EMLP29	8	3	Frozen, No preservative	98.4	99.2	0.991935484	25.8	25.59193548	1.319	1.58	0.66	17.6	11.72688	66.63	33.37	0.022949104	yes	yes
Larimichthys polyactis	EMLP33	9	3	Frozen, No preservative	108.1	107	1.010280374	26.4	26.67140187	0.607	3.3	0.64	18.6	11.7273	63.05	36.95	0.025768049	yes	yes
Larimichthys polyactis	EMLP37	10	3	Frozen, No preservative	92.9	90.1	1.031076582	28.2	29.0763596	1.98	1.8	1.43	131	108.0357	82.47	17.53	0.078979282	yes	no
Odontesthes regia	EMSS2	1	1	250 mM EDTA pH 10.0	101.9	179.5	0.567688022	24.8	14.07866295	0.323	2.57	0.93	19.7	13.80182	70.06	29.94	0.041894461	yes	yes
Odontesthes regia	EMSS6	2	1	250 mM EDTA pH 10.0	89.4	152.6	0.585845347	26.8	15.70065531	0.297	2.21	1.27	19.8	7.41708	37.46	62.54	0.07886881	yes	yes
Odontesthes regia	EMSS10	3	1	250 mM EDTA pH 10.0	106.5	117.6	0.905612245	20.9	18.92729592	0.356	2.45	1.24	27.2	21.4336	78.8	21.2	0.030466053	yes	yes
Odontesthes regia	EMSS14	4	1	250 mM EDTA pH 10.0	99	114.3	0.866141732	27	23.38582677	0.329	2.1	1.39	19.7	15.24189	77.37	22.63	0.019063299	yes	yes
Odontesthes regia	EMSS18	5	1	250 mM EDTA pH 10.0	105.3	99.1	1.062563068	24.7	26.24530777	0.26	2.65	0.73	15.9	11.9568	75.2	24.8	0.0150244	yes	yes
Odontesthes regia	EMSS22	6	1	250 mM EDTA pH 10.0	104.8	102.3	1.024437928	24.5	25.09872923	0.206	3.06	0.49	13.9	12.73796	91.64	8.36	0.004629876	yes	yes
Odontesthes regia	EMSS26	7	1	250 mM EDTA pH 10.0	96.5	84.6	1.140661939	21.9	24.98049645	0.32	2.6	0.91	22.6	14.5092	64.2	35.8	0.032388468	yes	yes
Odontesthes regia	EMSS30	8	1	250 mM EDTA pH 10.0	91.7	96.2	0.953222453	21.2	20.20831601	0.306	2.51	0.9	15.8	13.29886	84.17	15.83	0.012376786	yes	yes
Odontesthes regia	EMSS34	9	1	250 mM EDTA pH 10.0	91.3	74.4	1.227150538	28.5	34.97379032	0.204	2.87	0.69	15.4	14.25424	92.56	7.44	0.003276053	yes	yes
Odontesthes regia	EMSS38	10	1	250 mM EDTA pH 10.0	97.1	86.4	1.123842593	24	26.97222222	0.164	4	0.64	8.73	8.18874	93.8	6.2	0.002006731	yes	yes
Odontesthes regia	EMSS3	1	2	95% EtOH	92.6	69.8	1.326647564	24	31.83954155	0.412	2.32	1.2	25	20.5975	82.39	17.61	0.013827146	yes	yes
Odontesthes regia	EMSS7	2	2	95% EtOH	101.3	60.6	1.671617162	25.8	43.12772277	0.781	1.96	1.3	40.2	37.20108	92.54	7.46	0.006953578	yes	yes
Odontesthes regia	EMSS11	3	2	95% EtOH	90.5	54.5	1.660550459	26.5	44.00458716	1.7	1.71	0.97	46.2	42.73962	92.51	7.49	0.00786368	yes	yes
Odontesthes regia	EMSS15	4	2	95% EtOH	107.8	86.3	1.249130939	29.9	37.34901506	0.622	2.02	0.97	167	132.7984	79.52	20.48	0.091572964	yes	yes
Odontesthes regia	EMSS19	5	2	95% EtOH	90.5	64.7	1.398763524	27.1	37.9064915	1.699	1.72	0.87	34.6	32.2299	93.15	6.85	0.006252491	yes	yes
Odontesthes regia	EMSS23	6	2	95% EtOH	96.5	64.7	1.491499227	25.1	37.4366306	0.683	1.85	0.7	31.3	25.4782	81.4	18.6	0.015551079	yes	yes
Odontesthes regia	EMSS27	7	2	95% EtOH	97.7	67.2	1.453869048	22.1	32.13050595	0.57	1.99	0.64	28	25.5276	91.17	8.83	0.007694868	yes	yes
Odontesthes regia	EMSS31	8	2	95% EtOH	104.4	69	1.513043478	25.6	38.73391304	0.513	2	0.52	31.2	28.13616	90.18	9.82	0.007909968	yes	yes
Odontesthes regia	EMSS35	9	2	95% EtOH	106.8	63.4	1.684542587	28.7	48.34637224	0.427	2	0.78	16.5	15.34995	93.03	6.97	0.002378772	yes	yes
Odontesthes regia	EMSS39	10	2	95% EtOH	100	66.2	1.510574018	21.9	33.081571	0.806	1.94	1.01	42.6	38.05884	89.34	10.66	0.013727159	yes	yes
Odontesthes regia	EMSS1	1	3	Frozen, No preservative	102.6	100.6	1.019880716	25	25.49701789	0.491	1.75	0.67	17.7	15.36183	86.79	13.21	0.009170367	yes	yes
Odontesthes regia	EMSS5	2	3	Frozen, No preservative	94.7	92.8	1.020474138	21.1	21.53200431	0.301	2	2.67	19.6	17.39304	88.74	11.26	0.010249673	yes	yes
Odontesthes regia	EMSS9	3	3	Frozen, No preservative	95.4	94.9	1.005268704	20.7	20.80906217	0.393	2.17	3.1	21.8	20.62062	94.59	5.41	0.005667627	yes	yes
Odontesthes regia	EMSS13	4	3	Frozen, No preservative	101.4	101.2	1.001976285	25.6	25.65059289	0.424	2.26	4.01	23.1	21.61005	93.55	6.45	0.005808638	yes	yes
Odontesthes regia	EMSS17	5	3	Frozen, No preservative	95.9	95.5	1.004188482	25	25.10471204	0.161	2.53	2.86	9.63	8.895231	92.37	7.63	0.002926817	yes	yes
Odontesthes regia	EMSS21	6	3	Frozen, No preservative	94.4	90.1	1.04772475	28.2	29.54583796	0.267	2.34	7.27	12.5	12.00625	96.05	3.95	0.001671132	yes	yes
Odontesthes regia	EMSS25	7	3	Frozen, No preservative	96	93	1.032258065	28.8	29.72903226	0.287	2.35	3.47	11.1	10.58607	95.37	4.63	0.001728714	yes	yes
Odontesthes regia	EMSS29	8	3	Frozen, No preservative	100.2	98.5	1.017258883	25.8	26.24527919	0.368	1.93	1.93	22	19.4062	88.21	11.79	0.00988292	yes	yes
Odontesthes regia	EMSS33	9	3	Frozen, No preservative	97	97.4	0.995893224	29.2	29.08008214	0.27	2.67	2.14	11.6	10.79032	93.02	6.98	0.002784311	yes	yes
Odontesthes regia	EMSS37	10	3	Frozen, No preservative	92.3	87.4	1.056064073	21.8	23.0221968	0.108	4.19	12.97	5.92	5.642352	95.31	4.69	0.001206001	yes	yes
Sardina pilchardus	EMSP2	1	1	250 mM EDTA pH 10.0	97.8	166.2	0.588447653	33.6	19.77184116	0.318	2.38	1	17.8	4.29336	24.12	75.88	0.068312505	yes	yes
Sardina pilchardus	EMSP6	2	1	250 mM EDTA pH 10.0	100.9	157.3	0.64144946	30.9	19.8207883	0.959	1.74	0.75	36.3	11.35101	31.27	68.73	0.125872844	yes	yes
Sardina pilchardus	EMSP10	3	1	250 mM EDTA pH 10.0	103.2	180.8	0.57079646	26.3	15.0119469	0.37	1.75	0.43	9.8	2.0629	21.05	78.95	0.051539617	yes	yes
Sardina pilchardus	EMSP14	4	1	250 mM EDTA pH 10.0	102.7	167.9	0.611673615	29.1	17.7997022	0.068	4.19	0.43	6.24	2.166528	34.72	65.28	0.022885057	yes	yes
Sardina pilchardus	EMSP18	5	1	250 mM EDTA pH 10.0	100.2	142.4	0.703651685	24.8	17.4505618	0.64	1.73	0.49	10.6	2.3108	21.8	78.2	0.04750105	yes	yes
Sardina pilchardus	EMSP22	6	1	250 mM EDTA pH 10.0	108	172.2	0.6271777	30.6	19.19163763	0.903	1.83	0.72	27.7	8.69226	31.38	68.62	0.099041782	yes	yes
Sardina pilchardus	EMSP26	7	1	250 mM EDTA pH 10.0	109.7	157.5	0.696507937	25.9	18.03955556	0.872	1.46	0.52	8.07	2.063499	25.57	74.43	0.03329628	yes	yes
Sardina pilchardus	EMSP30	8	1	250 mM EDTA pH 10.0	104.1	169.6	0.61379717	34.7	21.29876179	0.305	2.12	1.03	12.9	2.93604	22.76	77.24	0.046781874	yes	yes
Sardina pilchardus	EMSP34	9	1	250 mM EDTA pH 10.0	90.3	125.4	0.720095694	32.3	23.25909091	0.175	1.71	0.28	5.11	1.575413	30.83	69.17	0.015196583	yes	yes
Sardina pilchardus	EMSP38	10	1	250 mM EDTA pH 10.0	91.2	152.2	0.599211564	39.9	23.90854139	0.297	1.69	0.34	8.88	2.426016	27.32	72.68	0.02699447	yes	yes
Sardina pilchardus	EMSP3	1	2	95% EtOH	109.6	68.4	1.602339181	33.6	53.83859649	0.415	1.81	0.22	3.73	2.054857	55.09	44.91	0.003111417	yes	yes
Sardina pilchardus	EMSP7	2	2	95% EtOH	108.2	72.5	1.492413793	27.5	41.04137931	0.397	1.89	0.2	3.75	1.930125	51.47	48.53	0.004434244	yes	yes
Sardina pilchardus	EMSP11	3	2	95% EtOH	105.8	80.4	1.315920398	30.7	40.39875622	1.3	1.63	0.33	6.54	2.769036	42.34	57.66	0.009334357	yes	yes
Sardina pilchardus	EMSP15	4	2	95% EtOH	107.3	77.4	1.38630491	21.6	29.94418605	0.302	2.01	0.3	8.66	2.08273	24.05	75.95	0.021965099	yes	yes
Sardina pilchardus	EMSP19	5	2	95% EtOH	106.8	72.7	1.469050894	24.6	36.13865199	0.94	1.58	0.41	34	12.1584	35.76	64.24	0.060438336	yes	yes
Sardina pilchardus	EMSP23	6	2	95% EtOH	106.1	75.9	1.397891963	28.2	39.42055336	0.822	1.51	0.28	14.3	8.3226	58.2	41.8	0.015163156	yes	yes
Sardina pilchardus	EMSP27	7	2	95% EtOH	98.9	73.7	1.34192673	24.7	33.14559023	0.749	1.61	0.47	20.1	7.92744	39.44	60.56	0.036724523	yes	yes
Sardina pilchardus	EMSP31	8	2	95% EtOH	100.9	65.2	1.547546012	27.1	41.93849693	0.38	1.79	0.18	5	2.233	44.66	55.34	0.006597757	yes	yes
Sardina pilchardus	EMSP35	9	2	95% EtOH	110.4	91.4	1.207877462	19.1	23.07045952	0.499	1.51	0.32	7.09	2.940932	41.48	58.52	0.017984332	yes	no
Sardina pilchardus	EMSP39	10	2	95% EtOH	96.1	77.4	1.241602067	31.6	39.23462532	2.967	1.8	1.25	83.9	55.82706	66.54	33.46	0.071551442	yes	yes
Sardina pilchardus	EMSP1	1	3	Frozen, No preservative	107.2	106.1	1.010367578	27.6	27.88614515	0.663	1.74	0.48	12.6	5.06772	40.22	59.78	0.027010833	yes	yes
Sardina pilchardus	EMSP5	2	3	Frozen, No preservative	102.4	100.7	1.016881827	33.4	33.96385303	0.874	1.64	0.49	27.2	16.72256	61.48	38.52	0.030848797	yes	yes
Sardina pilchardus	EMSP9	3	3	Frozen, No preservative	92.3	92.4	0.998917749	28.5	28.46915584	0.229	1.63	0.26	3.52	2.11552	60.1	39.9	0.004933339	yes	yes
Sardina pilchardus	EMSP13	4	3	Frozen, No preservative	97	94.8	1.023206751	27.5	28.13818565	0.556	1.86	0.62	18.7	7.92693	42.39	57.61	0.038286299	yes	yes
Sardina pilchardus	EMSP17	5	3	Frozen, No preservative	97.5	96.5	1.010362694	32.5	32.83678756	1.525	1.84	1.13	54.7	19.39115	35.45	64.55	0.107528332	yes	yes
Sardina pilchardus	EMSP21	6	3	Frozen, No preservative	102	100.4	1.015936255	38.5	39.11354582	0.36	1.77	0.22	8.78	3.436492	39.14	60.86	0.013661528	yes	yes
Sardina pilchardus	EMSP25	7	3	Frozen, No preservative	96.2	96	1.002083333	20.5	20.54270833	0.172	1.78	0.25	2.64	1.612248	61.07	38.93	0.005003001	yes	yes
Sardina pilchardus	EMSP29	8	3	Frozen, No preservative	105.4	102.9	1.024295432	31.6	32.36773567	0.462	1.58	0.37	4.49	1.750651	38.99	61.01	0.008463209	yes	yes
Sardina pilchardus	EMSP33	9	3	Frozen, No preservative	106.5	103.9	1.025024062	46.8	47.97112608	0.478	1.73	0.28	5.29	2.277874	43.06	56.94	0.00627904	yes	yes
Sardina pilchardus	EMSP37	10	3	Frozen, No preservative	105.8	104.3	1.014381592	34.1	34.59041227	3.062	1.53	0.35	46.3	28.00224	60.48	39.52	0.052898358	yes	yes
Amphioctopus aegina	EMOC6	1	1	250 mM EDTA pH 10.0	98	95.4	1.027253669	24.5	25.16771488	-0.171	1.3	0.29	4.02	3.813372	94.86	5.14	0.000821004	yes	yes
Amphioctopus aegina	EMOC10	2	1	250 mM EDTA pH 10.0	93.7	100.6	0.931411531	25.4	23.65785288	-0.097	0.97	0.29	9.06	8.435766	93.11	6.89	0.002638591	yes	yes
Amphioctopus aegina	EMOC14	3	1	250 mM EDTA pH 10.0	94.4	112.3	0.840605521	20.7	17.40053428	-0.176	1.22	0.6	2.41	2.203945	91.45	8.55	0.001184188	yes	yes
Amphioctopus aegina	EMOC18	4	1	250 mM EDTA pH 10.0	103.3	137.7	0.750181554	28.6	21.45519245	-0.173	0.98	0.59	2.24	2.067968	92.32	7.68	0.00080182	yes	yes
Amphioctopus aegina	EMOC22	5	1	250 mM EDTA pH 10.0	95.4	113.9	0.837576822	25.8	21.609482	-0.106	0.91	0.49	5.99	5.728836	95.64	4.36	0.001208562	yes	yes
Amphioctopus aegina	EMOC26	6	1	250 mM EDTA pH 10.0	95.9	105.8	0.906427221	29.7	26.92088847	-0.128	1.04	-0.98	3.1	2.64244	85.24	14.76	0.001699647	yes	yes
Amphioctopus fangsiao	EMOC30	7	1	250 mM EDTA pH 10.0	108.8	97.7	1.113613101	24.6	27.39488229	-0.078	0.83	0.17	5.3	5.09595	96.15	3.85	0.000744847	yes	no
Amphioctopus aegina	EMOC34	8	1	250 mM EDTA pH 10.0	97.7	97.7	1	25.5	25.5	-0.07	0.64	0.23	5.83	5.249915	90.05	9.95	0.002274843	yes	yes
Amphioctopus fangsiao	EMOC38	9	1	250 mM EDTA pH 10.0	106	127.5	0.831372549	33.2	27.60156863	0.184	4.68	-0.68	18.5	17.06625	92.25	7.75	0.005194451	yes	yes
Amphioctopus aegina	EMOC42	10	1	250 mM EDTA pH 10.0	107.4	107.5	0.999069767	25.6	25.57618605	0.122	0.04	0.54	4.48	4.30752	96.15	3.85	0.000674377	yes	yes
Amphioctopus aegina	EMOC7	1	2	95% EtOH	101.6	67.3	1.509658247	33.4	50.42258544	0.022	-0.65	-0.13	2.92	2.629752	90.06	9.94	0.000575631	yes	yes
Amphioctopus aegina	EMOC11	2	2	95% EtOH	92.8	58.4	1.589041096	26.9	42.74520548	0.218	3.56	-0.77	2.61	2.480283	95.03	4.97	0.000303466	yes	yes
Amphioctopus aegina	EMOC15	3	2	95% EtOH	109.9	71.8	1.530640669	22.2	33.98022284	0.488	2.03	1.12	4.69	4.365452	93.08	6.92	0.000955109	no	no
Amphioctopus aegina	EMOC19	4	2	95% EtOH	100.6	63.5	1.584251969	26.6	42.14110236	0.824	1.91	0.72	2.46	2.205144	89.64	10.36	0.000604768	no	no
Amphioctopus aegina	EMOC23	5	2	95% EtOH	89.1	61.1	1.458265139	20.8	30.33191489	0.089	-7	0.97	2.36	2.104884	89.19	10.81	0.000841081	yes	no
Amphioctopus aegina	EMOC27	6	2	95% EtOH	100.6	54.3	1.85267035	32.9	60.95285451	-0.024	0.37	0.11	3.09	2.473854	80.06	19.94	0.001010857	yes	yes

Species	Sample ID	Specimen	Replicate	Treatment	Initial Sample Weight (mg)	Post-treatment Sample Weight (mg)	Correction Ratio	Weight of Tissue Used for Extraction (mg)	Corrected Weight of Tissue Used for Extraction (mg)	A_{260}	A_{260}/A_{280} Ratio	A_{260}/A_{230} Ratio	Total DNA Concentration (ng/µL)	LMW DNA Concentration (ng/µL)	%LMW	%HMW	nY (µg DNA/mg tissue)	*COI* PCR Amplification Success	*COI* Sequencing Success
Amphioctopus fangsiao	EMOC31	7	2	95% EtOH	100.3	67.8	1.479351032	35.7	52.81283186	0.038	-0.85	-0.13	3.42	3.095784	90.52	9.48	0.000613896	yes	yes
Amphioctopus aegina	EMOC35	8	2	95% EtOH	96.9	54.5	1.777981651	28.1	49.9612844	0.02	-0.45	-0.21	3.13	2.666134	85.18	14.82	0.000928451	yes	no
Amphioctopus fangsiao	EMOC39	9	2	95% EtOH	92.6	51.1	1.812133072	31	56.17612524	0.011	-0.27	-0.05	11.1	10.54278	94.98	5.02	0.000991916	yes	yes
Amphioctopus aegina	EMOC43	10	2	95% EtOH	110.6	76.3	1.449541284	25.9	37.54311927	0.381	0.181	0.52	2.24	2.041088	91.12	8.88	0.000529823	no	yes
Amphioctopus aegina	EMOC5	1	3	Frozen, No preservative	107.5	103.8	1.035645472	24.8	25.68400771	0.076	-4.19	1.6	1.82	1.51242	83.1	16.9	0.001197555	no	no
Amphioctopus aegina	EMOC9	2	3	Frozen, No preservative	100.4	93.5	1.073796791	30.4	32.64342246	0.136	9.64	-0.74	10.1	9.13545	90.45	9.55	0.002954807	yes	yes
Amphioctopus aegina	EMOC13	3	3	Frozen, No preservative	97.2	93.8	1.036247335	19.9	20.62132196	-0.124	1.08	0.31	3.2	2.9152	91.1	8.9	0.001381095	yes	yes
Amphioctopus aegina	EMOC17	4	3	Frozen, No preservative	103.2	100.5	1.026865672	25.4	26.08238806	0.053	-2.44	-2.44	2.58	2.317356	89.82	10.18	0.001006978	no	no
Amphioctopus aegina	EMOC21	5	3	Frozen, No preservative	105.7	102.9	1.027210884	17.5	17.97619048	-0.112	0.88	0.36	1.66	1.524876	91.86	8.14	0.000751683	yes	yes
Amphioctopus aegina	EMOC25	6	3	Frozen, No preservative	90.2	89.2	1.011210762	26.1	26.3926009	-0.126	0.98	0.28	1.98	1.739826	87.87	12.13	0.000910005	yes	yes
Amphioctopus fangsiao	EMOC29	7	3	Frozen, No preservative	100.2	103.9	0.964388835	25.5	24.5919153	0.205	3.2	-0.85	16.1	15.24831	94.71	5.29	0.003463293	yes	yes
Amphioctopus aegina	EMOC33	8	3	Frozen, No preservative	96.8	84.6	1.144208038	32	36.61465721	0.422	1.96	0.88	5.17	4.809651	93.03	6.97	0.000984166	no	no
Amphioctopus fangsiao	EMOC37	9	3	Frozen, No preservative	95.8	91.8	1.043572985	20.3	21.18453159	-0.183	1.11	0.53	5.73	5.335203	93.11	6.89	0.00186361	yes	yes
Amphioctopus aegina	EMOC41	10	3	Frozen, No preservative	110.7	103.2	1.072674419	27.1	29.06947674	0.161	0.041	0.29	2.87	2.649297	92.31	7.69	0.000759226	yes	yes
Homarus americanus	YBOL21	1	1	250 mM EDTA pH 10.0	107.5	n/a	1	24.9	24.9	0.476	1.84	0.52	10.3	6.13262	59.54	40.46	0.016736466	yes	yes
Homarus americanus	YBOL22	2	1	250 mM EDTA pH 10.0	107.3	n/a	1	23.4	23.4	0.452	1.95	0.64	12.1	8.3611	69.1	30.9	0.015978205	yes	yes
Homarus americanus	YBOL23	3	1	250 mM EDTA pH 10.0	103.9	n/a	1	21.8	21.8	0.401	1.71	0.43	1.76	1.366464	77.64	22.36	0.001805211	yes	yes
Homarus americanus	YBOL24	4	1	250 mM EDTA pH 10.0	98.4	n/a	1	29.1	29.1	0.496	1.61	0.54	9.6	4.23456	44.11	55.89	0.018437938	yes	yes
Homarus americanus	YBOL25	5	1	250 mM EDTA pH 10.0	111.8	n/a	1	25.3	25.3	0.178	1.62	0.61	5.31	2.07621	39.1	60.9	0.012781779	yes	yes
Homarus americanus	YBOL26	6	1	250 mM EDTA pH 10.0	106.4	n/a	1	23.2	23.2	0.205	2.37	1.88	61.2	31.13244	50.87	49.13	0.129601552	yes	yes
Homarus americanus	YBOL27	7	1	250 mM EDTA pH 10.0	109.1	n/a	1	26.5	26.5	0.388	1.7	0.53	4.13	2.71754	65.8	34.2	0.005330038	yes	yes
Homarus americanus	YBOL28	8	1	250 mM EDTA pH 10.0	114.1	n/a	1	24.4	24.4	0.755	1.6	0.46	7.18	3.258284	45.38	54.62	0.016072607	yes	yes
Homarus americanus	YBOL29	9	1	250 mM EDTA pH 10.0	96.5	n/a	1	23.8	23.8	0.521	1.62	0.36	7.15	4.033315	56.41	43.59	0.013095315	yes	yes
Homarus americanus	YBOL30	10	1	250 mM EDTA pH 10.0	108.7	n/a	1	25.7	25.7	0.351	1.56	0.35	3.02	1.166022	38.61	61.39	0.007213922	yes	yes
Homarus americanus	YBOL31	1	2	95% EtOH	97.1	n/a	1	28.3	28.3	0.618	1.77	0.7	73.3	47.0586	64.2	35.8	0.092725795	yes	yes
Homarus americanus	YBOL32	2	2	95% EtOH	102.8	n/a	1	26.9	26.9	0.433	1.66	0.54	8.15	7.731905	94.87	5.13	0.001554257	yes	yes
Homarus americanus	YBOL33	3	2	95% EtOH	96.9	n/a	1	27.9	27.9	0.688	1.55	0.55	14.2	12.81976	90.28	9.72	0.004947097	yes	yes
Homarus americanus	YBOL34	4	2	95% EtOH	100	n/a	1	24.6	24.6	0.623	1.81	0.67	26.7	23.15157	86.71	13.29	0.014424512	yes	yes
Homarus americanus	YBOL35	5	2	95% EtOH	106.5	n/a	1	23.8	23.8	0.801	1.68	0.81	23.2	15.15888	65.34	34.66	0.033786218	yes	yes
Homarus americanus	YBOL36	6	2	95% EtOH	108.5	n/a	1	24.8	24.8	0.55	1.6	0.49	13.1	8.07746	61.66	38.34	0.020252177	yes	yes
Homarus americanus	YBOL37	7	2	95% EtOH	102.4	n/a	1	26.6	26.6	0.549	1.71	0.51	15.6	10.90752	69.92	30.08	0.017640902	yes	yes
Homarus americanus	YBOL38	8	2	95% EtOH	114.4	n/a	1	22.6	22.6	0.641	1.9	0.49	25.7	17.54025	68.25	31.75	0.036105088	yes	yes
Homarus americanus	YBOL39	9	2	95% EtOH	112.6	n/a	1	23.5	23.5	0.703	1.8	0.67	16.2	12.57444	77.62	22.38	0.015427915	yes	yes
Homarus americanus	YBOL40	10	2	95% EtOH	106.6	n/a	1	22.6	22.6	1.405	1.69	0.82	44	29.4536	66.94	33.06	0.064364602	yes	yes
Homarus americanus	YBOL1	1	3	Frozen, No preservative	23.5	n/a	1	23.5	23.5	0.259	2.15	1.24	7.48	4.597208	61.46	38.54	0.0122672	yes	yes
Homarus americanus	YBOL2	2	3	Frozen, No preservative	22.7	n/a	1	22.7	22.7	0.204	1.91	1.14	15.9	14.46741	90.99	9.01	0.006310969	yes	yes
Homarus americanus	YBOL3	3	3	Frozen, No preservative	26.1	n/a	1	26.1	26.1	0.26	2.69	1.27	15.7	14.80196	94.28	5.72	0.003440766	yes	yes
Homarus americanus	YBOL4	4	3	Frozen, No preservative	25.6	n/a	1	25.6	25.6	0.559	1.89	1.85	30.2	24.87574	82.37	17.63	0.020797891	yes	yes
Homarus americanus	YBOL5	5	3	Frozen, No preservative	24.9	n/a	1	24.9	24.9	0.192	2.27	0.67	4	2.6876	67.19	32.81	0.005270683	yes	yes
Homarus americanus	YBOL6	6	3	Frozen, No preservative	25.2	n/a	1	25.2	25.2	0.213	2.43	0.85	11.9	9.5319	80.1	19.9	0.009397222	yes	yes
Homarus americanus	YBOL7	7	3	Frozen, No preservative	26.6	n/a	1	26.6	26.6	0.33	2.23	0.98	17	13.5473	79.69	20.31	0.012980075	yes	yes
Homarus americanus	YBOL8	8	3	Frozen, No preservative	25.1	n/a	1	25.1	25.1	0.569	1.99	1.71	35	29.6205	84.63	15.37	0.021432271	yes	yes
Homarus americanus	YBOL9	9	3	Frozen, No preservative	25.5	n/a	1	25.5	25.5	0.198	2.4	0.66	10.8	8.51688	78.86	21.14	0.008953412	yes	yes
Homarus americanus	YBOL10	10	3	Frozen, No preservative	27.3	n/a	1	27.3	27.3	0.292	2.06	1.13	19.2	16.64832	86.71	13.29	0.009346813	yes	yes
Magallana gigas	EMOY2	1	1	250 mM EDTA pH 10.0	102.8	77.8	1.321336761	22.6	29.8622108	3.823	2.174	0.93	152	62.0616	40.83	59.17	0.301177969	yes	yes
Magallana gigas	EMOY6	2	1	250 mM EDTA pH 10.0	96.6	98.2	0.983706721	21.9	21.54317719	2.825	1.59	0.9	83.8	37.8776	45.2	54.8	0.213164472	yes	yes
Magallana gigas	EMOY10	3	1	250 mM EDTA pH 10.0	93	79.8	1.165413534	22.5	26.22180451	2.135	1.235	0.87	51.7	26.04646	50.38	49.62	0.097832855	yes	yes
Magallana gigas	EMOY14	4	1	250 mM EDTA pH 10.0	93	100.2	0.928143713	21.4	19.86227545	3.186	1.878	0.81	93.2	61.17648	65.64	34.36	0.161227852	yes	yes
Magallana gigas	EMOY18	5	1	250 mM EDTA pH 10.0	94.4	99.2	0.951612903	24.3	23.12419355	3.497	2.037	0.93	41.3	9.75093	23.61	76.39	0.136433169	yes	yes
Magallana gigas	EMOY22	6	1	250 mM EDTA pH 10.0	96.8	90.8	1.066079295	27.3	29.10396476	2.24	1.267	0.9	46.8	35.39952	75.64	24.36	0.03917157	yes	yes
Magallana gigas	EMOY26	7	1	250 mM EDTA pH 10.0	93.2	113.5	0.821145374	26.9	22.08881057	2.624	1.444	1.02	122	95.0746	77.93	22.07	0.121896106	yes	yes
Magallana gigas	EMOY30	8	1	250 mM EDTA pH 10.0	95.8	95.9	0.998957247	22.9	22.87612096	3.453	2.016	0.85	82.5	41.88525	50.77	49.23	0.177542119	yes	yes
Magallana gigas	EMOY34	9	1	250 mM EDTA pH 10.0	98.8	125.1	0.789768185	29.2	23.06123102	1.012	0.531	1.15	31.6	21.1246	66.85	33.15	0.045424288	yes	yes
Magallana gigas	EMOY38	10	1	250 mM EDTA pH 10.0	107.5	155.1	0.693101225	20.8	14.41650548	2.364	1.359	0.81	68.7	27.08841	39.43	60.57	0.288638533	yes	yes
Magallana gigas	EMOY3	1	2	95% EtOH	92.7	60.7	1.527182867	28.3	43.21927512	4.229	2.404	0.81	73	62.8968	86.16	13.84	0.023376607	yes	yes
Magallana gigas	EMOY7	2	2	95% EtOH	106.9	70.7	1.512022631	17.6	26.6115983	2.721	1.544	0.78	28.9	25.36553	87.77	12.23	0.01328169	yes	yes
Magallana gigas	EMOY11	3	2	95% EtOH	99.2	60.9	1.628899836	26.9	43.81740558	1.928	1.147	0.64	27.5	23.881	86.84	13.16	0.008259275	yes	yes
Magallana gigas	EMOY15	4	2	95% EtOH	101.4	77.2	1.313471503	27	35.46373057	1.94	1.126	0.84	31.4	28.05276	89.34	10.66	0.009438488	yes	yes
Magallana gigas	EMOY19	5	2	95% EtOH	107.4	66.1	1.624810893	24.5	39.80786687	4.358	2.49	0.69	70.5	57.92985	82.17	17.83	0.03157705	yes	yes
Magallana gigas	EMOY23	6	2	95% EtOH	94	57.9	1.623488774	28.3	45.9447323	2.247	1.254	0.85	29.4	26.63346	90.59	9.41	0.006021452	yes	yes
Magallana gigas	EMOY27	7	2	95% EtOH	99.4	68.3	1.45534407	22.8	33.1818448	n/a	n/a	n/a	31.9	30.31457	95.03	4.97	0.004778004	yes	yes
Magallana gigas	EMOY31	8	2	95% EtOH	91	53.2	1.710526316	24.8	42.42105263	3.363	1.916	0.83	46.6	41.008	88	12	0.013182134	yes	yes
Magallana gigas	EMOY35	9	2	95% EtOH	105.7	70.5	1.49929078	24.4	36.58269504	0.687	0.375	0.36	16.1	13.72364	85.24	14.76	0.006495858	yes	yes
Magallana gigas	EMOY39	10	2	95% EtOH	106.1	66.1	1.605143722	24.1	38.68396369	5.706	3.237	0.94	134	112.2384	83.76	16.24	0.056254835	yes	yes
Magallana gigas	EMOY1	1	3	Frozen, No preservative	95	89.3	1.063829787	24.5	26.06382979	0.857	0.487	0.55	12.1	8.93585	73.85	26.15	0.012140004	yes	yes
Magallana gigas	EMOY5	2	3	Frozen, No preservative	108.4	107.1	1.012138189	26.3	26.61923436	2.303	1.314	0.59	12.9	9.54729	74.01	25.99	0.012595065	yes	yes
Magallana gigas	EMOY9	3	3	Frozen, No preservative	92.6	87.3	1.060710195	24.3	25.77525773	0.849	0.434	0.61	17.8	12.71632	71.44	28.56	0.019723101	yes	yes
Magallana gigas	EMOY13	4	3	Frozen, No preservative	99.1	93.2	1.063304721	29.6	31.47381974	1.505	0.878	0.63	24.2	17.22072	71.16	28.84	0.022174874	yes	yes
Magallana gigas	EMOY17	5	3	Frozen, No preservative	109.2	103.3	1.057115198	19.4	20.50803485	1.108	0.645	0.8	16.7	13.61885	81.55	18.45	0.015024111	yes	yes
Magallana gigas	EMOY21	6	3	Frozen, No preservative	101.6	86.7	1.171856978	26.3	30.81983852	1.259	0.72	0.69	12.6	9.66294	76.69	23.31	0.009529771	yes	yes
Magallana gigas	EMOY25	7	3	Frozen, No preservative	95.2	88.7	1.073280722	28.8	30.91048478	2.077	1.175	0.72	11.4	10.00236	87.74	12.26	0.004521573	yes	yes
Magallana gigas	EMOY29	8	3	Frozen, No preservative	99.6	92.1	1.081433225	20.3	21.95309446	1.232	0.663	0.74	17.7	14.27859	80.67	19.33	0.015585092	yes	yes
Magallana gigas	EMOY33	9	3	Frozen, No preservative	88	85.3	1.031652989	24.1	24.86283705	3.368	1.859	1.05	86	73.9342	85.97	14.03	0.048529458	yes	yes
Magallana gigas	EMOY37	10	3	Frozen, No preservative	105.2	100.1	1.050949051	25.2	26.48391608	2.872	1.597	1.05	65.5	55.3606	84.52	15.48	0.038285124	yes	yes
Mercenaria mercenaria	YBC21	1	1	250 mM EDTA pH 10.0	94.2	n/a	1	24.2	24.2	0.817	1.55	0.43	8.06	3.576222	44.37	55.63	0.018528008	yes	yes
Mercenaria mercenaria	YBC22	2	1	250 mM EDTA pH 10.0	102.4	n/a	1	25.5	25.5	1.052	1.56	0.45	10	4.933	49.33	50.67	0.019870588	yes	yes
Mercenaria mercenaria	YBC23	3	1	250 mM EDTA pH 10.0	99.5	n/a	1	26.1	26.1	0.641	1.65	0.46	18.3	7.10955	38.85	61.15	0.042875287	yes	yes
Mercenaria mercenaria	YBC24	4	1	250 mM EDTA pH 10.0	99.6	n/a	1	24.3	24.3	0.623	1.53	0.37	5.12	1.85344	36.2	63.8	0.013442634	yes	yes
Mercenaria mercenaria	YBC25	5	1	250 mM EDTA pH 10.0	108.5	n/a	1	27.2	27.2	0.438	1.56	0.4	5.99	2.488246	41.54	58.46	0.012874096	yes	yes
Mercenaria mercenaria	YBC26	6	1	250 mM EDTA pH 10.0	69.5	n/a	1	25.6	25.6	0.25	1.65	0.44	4.5	2.1483	47.74	52.26	0.009186328	yes	yes
Mercenaria mercenaria	YBC27	7	1	250 mM EDTA pH 10.0	58.4	n/a	1	27.5	27.5	0.915	1.53	0.47	10.6	3.16304	29.84	70.16	0.027043491	yes	yes
Mercenaria mercenaria	YBC28	8	1	250 mM EDTA pH 10.0	103.1	n/a	1	25	25	0.978	1.59	0.46	8.04	2.9346	36.5	63.5	0.0204216	yes	yes
Mercenaria mercenaria	YBC29	9	1	250 mM EDTA pH 10.0	110.2	n/a	1	27.2	27.2	1.06	1.58	0.46	11.8	2.9146	24.7	75.3	0.032666912	yes	yes

Species	Sample ID	Specimen	Replicate	Treatment	Initial Sample Weight (mg)	Post-treatment Sample Weight (mg)	Correction Ratio	Weight of Tissue Used for Extraction (mg)	Corrected Weight of Tissue Used for Extraction (mg)	A_{260}	A_{260}/A_{280} Ratio	A_{260}/A_{230} Ratio	Total DNA Concentration (ng/µL)	LMW DNA Concentration (ng/µL)	%LMW	%HMW	nY (µg DNA/mg tissue)	*COI* PCR Amplification Success	*COI* Sequencing Success
Mercenaria mercenaria	YBC30	10	1	250 mM EDTA pH 10.0	103.9	n/a	1	25.1	25.1	0.697	1.55	0.41	5.14	2.100718	40.87	59.13	0.012108693	yes	yes
Mercenaria mercenaria	YBC31	1	2	95% EtOH	108.3	n/a	1	26.8	26.8	3.281	1.58	0.57	9.62	4.629144	48.12	51.88	0.018622597	yes	yes
Mercenaria mercenaria	YBC32	2	2	95% EtOH	102.6	n/a	1	26.6	26.6	1.383	1.65	0.37	141	68.8221	48.81	51.19	0.271345489	yes	yes
Mercenaria mercenaria	YBC33	3	2	95% EtOH	106.9	n/a	1	25.9	25.9	1.167	1.63	0.41	7.33	2.535447	34.59	65.41	0.018511788	yes	yes
Mercenaria mercenaria	YBC34	4	2	95% EtOH	108.8	n/a	1	26.6	26.6	0.952	1.61	0.39	27	11.2104	41.52	58.48	0.059359398	yes	yes
Mercenaria mercenaria	YBC35	5	2	95% EtOH	104.9	n/a	1	25.3	25.3	8.428	1.58	0.75	69.7	37.92377	54.41	45.59	0.125597747	yes	yes
Mercenaria mercenaria	YBC36	6	2	95% EtOH	39.1	n/a	1	24.7	24.7	1.687	1.6	0.46	54.4	26.47104	48.66	51.34	0.113072713	yes	no
Mercenaria mercenaria	YBC37	7	2	95% EtOH	51.1	n/a	1	26.3	26.3	0.789	1.53	0.32	9.38	2.73896	29.2	70.8	0.025251103	yes	yes
Mercenaria mercenaria	YBC38	8	2	95% EtOH	102.3	n/a	1	23.9	23.9	5.309	1.56	0.61	16.6	5.73364	34.54	65.46	0.045465941	yes	yes
Mercenaria mercenaria	YBC39	9	2	95% EtOH	102.4	n/a	1	27.6	27.6	1.018	1.67	0.4	24.1	8.77722	36.42	63.58	0.055517319	yes	yes
Mercenaria mercenaria	YBC40	10	2	95% EtOH	107.4	n/a	1	26.5	26.5	0.793	1.5	0.32	5.15	2.1218	41.2	58.8	0.01142717	yes	yes
Mercenaria mercenaria	YBC1	1	3	Frozen, No preservative	23.6	n/a	1	23.6	23.6	1.066	1.54	0.33	7.5	5.6895	75.86	24.14	0.00767161	yes	yes
Mercenaria mercenaria	YBC2	2	3	Frozen, No preservative	26	n/a	1	26	26	1.704	1.64	0.44	175	150.01	85.72	14.28	0.096115385	yes	yes
Mercenaria mercenaria	YBC3	3	3	Frozen, No preservative	25.3	n/a	1	25.3	25.3	1.762	1.57	0.37	6.07	4.008021	66.03	33.97	0.008150115	yes	yes
Mercenaria mercenaria	YBC4	4	3	Frozen, No preservative	24	n/a	1	24	24	1.518	1.53	0.32	8.02	5.050194	62.97	37.03	0.012374192	yes	yes
Mercenaria mercenaria	YBC5	5	3	Frozen, No preservative	27.8	n/a	1	27.8	27.8	0.934	1.67	0.32	23.6	17.58908	74.53	25.47	0.021622014	yes	yes
Mercenaria mercenaria	YBC6	6	3	Frozen, No preservative	23.5	n/a	1	23.5	23.5	1.954	1.54	0.43	13.4	9.63594	71.91	28.09	0.016017277	yes	yes
Mercenaria mercenaria	YBC7	7	3	Frozen, No preservative	26.3	n/a	1	26.3	26.3	1.752	1.6	0.51	30.6	10.51416	34.36	65.64	0.076372015	yes	yes
Mercenaria mercenaria	YBC8	8	3	Frozen, No preservative	27.7	n/a	1	27.7	27.7	2.422	1.6	0.45	22.3	11.91266	53.42	46.58	0.037499422	yes	yes
Mercenaria mercenaria	YBC9	9	3	Frozen, No preservative	25.2	n/a	1	25.2	25.2	2.301	1.59	0.39	23.6	17.0864	72.4	27.6	0.025847619	yes	yes
Mercenaria mercenaria	YBC10	10	3	Frozen, No preservative	25.7	n/a	1	25.7	25.7	0.959	1.49	0.3	13	8.4747	65.19	34.81	0.017608171	yes	yes
Penaeus vannamei	EMSH2	1	1	250 mM EDTA pH 10.0	103.7	152.8	0.678664921	23.7	16.08435864	0.052	63.09	0.24	6.12	2.668932	43.61	56.39	0.02145605	yes	yes
Penaeus vannamei	EMSH6	2	1	250 mM EDTA pH 10.0	99	146.2	0.677154583	26	17.60601915	-0.139	1.02	0.82	4.03	2.15605	53.5	46.5	0.010643803	yes	yes
Penaeus vannamei	EMSH10	3	1	250 mM EDTA pH 10.0	95.3	142.8	0.667366947	21.7	14.48186275	-0.077	0.76	0.35	8.82	3.331314	37.77	62.23	0.037900414	yes	yes
Penaeus vannamei	EMSH14	4	1	250 mM EDTA pH 10.0	94.5	133.2	0.709459459	28.8	20.43243243	-0.027	0.64	0.19	6.93	3.573801	51.57	48.43	0.016425842	yes	yes
Penaeus vannamei	EMSH18	5	1	250 mM EDTA pH 10.0	91.4	147.3	0.620502376	27.2	16.87766463	0.428	1.86	0.46	7.51	2.990482	39.82	60.18	0.026778101	yes	yes
Penaeus vannamei	EMSH22	6	1	250 mM EDTA pH 10.0	108.4	169.3	0.64028352	20.2	12.93372711	0.073	-3.2	-13.69	11.5	3.8479	33.46	66.54	0.05916392	yes	yes
Penaeus vannamei	EMSH26	7	1	250 mM EDTA pH 10.0	106.5	156.6	0.680076628	29.1	19.79022989	0.753	1.81	1.47	26.1	15.61563	59.83	40.17	0.052977505	yes	yes
Penaeus vannamei	EMSH30	8	1	250 mM EDTA pH 10.0	98.7	154.2	0.640077821	26.6	17.02607004	-0.086	0.96	0.35	8.89	4.694809	52.81	47.19	0.024639808	yes	yes
Penaeus vannamei	EMSH34	9	1	250 mM EDTA pH 10.0	101.5	145	0.7	25.3	17.71	0.787	1.71	0.69	19.2	6.81216	35.48	64.52	0.069948278	yes	yes
Penaeus vannamei	EMSH38	10	1	250 mM EDTA pH 10.0	112.4	174.6	0.643757159	28.2	18.15395189	-0.064	0.81	0.25	5.19	2.135685	41.15	58.85	0.016824519	yes	yes
Penaeus vannamei	EMSH3	1	2	95% EtOH	103.1	76.2	1.353018373	23.8	32.20183727	-0.069	0.93	-2.08	6.87	3.588201	52.23	47.77	0.010191341	yes	yes
Penaeus vannamei	EMSH7	2	2	95% EtOH	107.5	82.4	1.30461165	26.5	34.57220874	-0.07	0.69	0.68	7.25	3.999825	55.17	44.83	0.00940112	yes	yes
Penaeus vannamei	EMSH11	3	2	95% EtOH	93.4	65.5	1.425954198	26.6	37.93038168	-0.052	0.58	0.42	8.57	4.325279	50.47	49.53	0.011190821	yes	yes
Penaeus vannamei	EMSH15	4	2	95% EtOH	113	81.2	1.391625616	25.9	36.04310345	-0.071	0.74	-0.57	4.77	2.420775	50.75	49.25	0.006517821	yes	yes
Penaeus vannamei	EMSH19	5	2	95% EtOH	92.2	59.3	1.554806071	28.3	44.0010118	-0.041	0.72	-0.11	3.72	2.367408	63.64	36.36	0.003074002	yes	yes
Penaeus vannamei	EMSH23	6	2	95% EtOH	98.5	64.7	1.522411128	24.1	36.69010819	0.014	-0.44	-0.19	8.15	4.701735	57.69	42.31	0.009398351	yes	yes
Penaeus vannamei	EMSH27	7	2	95% EtOH	109.1	71	1.536619718	29	44.56197183	0.086	108.4	0.11	12.7	7.30885	57.55	42.45	0.012098096	yes	yes
Penaeus vannamei	EMSH31	8	2	95% EtOH	102.5	72.3	1.417704011	22.7	32.18188105	-0.002	0.04	-1.01	10.3	5.01301	48.67	51.33	0.016428468	yes	yes
Penaeus vannamei	EMSH35	9	2	95% EtOH	106.6	65.8	1.62006079	25.6	41.47355623	0.133	8.45	0.16	9.09	6.248466	68.74	31.26	0.006851436	yes	yes
Penaeus vannamei	EMSH39	10	2	95% EtOH	104.8	65.1	1.609831029	21.3	34.28940092	0.436	1.97	0.82	24.4	5.89992	24.18	75.82	0.053952765	yes	yes
Penaeus vannamei	EMSH1	1	3	Frozen, No preservative	92.4	90.3	1.023255814	24.3	24.86511628	-0.087	1	420.03	3.57	1.894242	53.06	46.94	0.006739393	yes	yes
Penaeus vannamei	EMSH5	2	3	Frozen, No preservative	106.7	106.2	1.004708098	20	20.09416196	-0.103	0.94	0.66	3.28	1.794816	54.72	45.28	0.007391122	yes	yes
Penaeus vannamei	EMSH9	3	3	Frozen, No preservative	107.9	105.9	1.018885741	24.3	24.75892351	-0.063	0.64	0.33	8.82	4.137462	46.91	53.09	0.018912527	yes	yes
Penaeus vannamei	EMSH13	4	3	Frozen, No preservative	92.6	90.9	1.01870187	28.5	29.0330033	0.067	-9.47	0.42	6.16	3.155152	51.22	48.78	0.010349766	yes	yes
Penaeus vannamei	EMSH17	5	3	Frozen, No preservative	112.2	110.5	1.015384615	24.7	25.08	-0.133	1.11	0.44	4.65	2.24502	48.28	51.72	0.009589234	yes	yes
Penaeus vannamei	EMSH21	6	3	Frozen, No preservative	109.1	108	1.010185185	24.4	24.64851852	0.239	2.64	0.52	11.2	4.36016	38.93	61.07	0.027749497	yes	yes
Penaeus vannamei	EMSH25	7	3	Frozen, No preservative	106.2	105	1.011428571	25.1	25.38685714	0.063	-3.85	-0.29	15.6	6.03252	38.67	61.33	0.037686745	yes	yes
Penaeus vannamei	EMSH29	8	3	Frozen, No preservative	100	98.5	1.015228426	19.4	19.69543147	-0.098	0.79	0.33	6.68	3.61722	54.15	45.85	0.015550713	yes	yes
Penaeus vannamei	EMSH33	9	3	Frozen, No preservative	94.9	91.8	1.033769063	30.3	31.32320261	0.692	1.73	0.98	31.2	13.53144	43.37	56.63	0.056407259	yes	yes
Penaeus vannamei	EMSH37	10	3	Frozen, No preservative	103.6	102.2	1.01369863	27.7	28.07945205	-0.127	1.16	0.36	4.53	2.877003	63.51	36.49	0.005886856	yes	yes
Alosa mediocris	EMAM2	1	1	250 mM EDTA pH 10.0	97.1	229.2	0.423647469	29.3	12.41287086	0.072	5.63	-0.16	16.5	4.52595	27.43	72.57	0.096464792	yes	no
Alosa mediocris	EMAM6	2	1	250 mM EDTA pH 10.0	98.1	211.8	0.463172805	28	12.96883853	0.027	-6.49	-0.1	9.26	3.715112	40.12	59.88	0.042755471	yes	yes
Alosa mediocris	EMAM10	3	1	250 mM EDTA pH 10.0	102.3	245.3	0.417040359	24	10.00896861	0.13	1.64	1.68	4.52	2.51538	55.65	44.35	0.020028237	yes	yes
Alosa mediocris	EMAM3	1	2	95% EtOH	98.6	79.4	1.241813602	21	26.07808564	0.363	1.55	0.19	4.22	2.952734	69.97	30.03	0.004859505	yes	yes
Alosa mediocris	EMAM7	2	2	95% EtOH	102.1	84.8	1.204009434	23.3	28.05341981	0.358	1.88	10.03	8.74	4.472258	51.17	48.83	0.015212912	yes	yes
Alosa mediocris	EMAM11	3	2	95% EtOH	96.8	93.8	1.031982942	22.2	22.91002132	0.111	2.17	0.9	3.02	2.368586	78.43	21.57	0.002843358	yes	yes
Alosa mediocris	EMAM1	1	3	Frozen, No preservative	103.1	103.4	0.997098646	23.6	23.53152805	0.064	5.65	-0.16	13.6	5.94864	43.74	56.26	0.032515356	yes	yes
Alosa mediocris	EMAM5	2	3	Frozen, No preservative	100.3	111.4	0.900359066	23.9	21.51858169	0.088	2.36	-0.33	9.31	4.920335	52.85	47.15	0.020399416	yes	yes
Alosa mediocris	EMAM9	3	3	Frozen, No preservative	104	100.3	1.036889332	21.4	22.1894317	-0.076	1.51	0.18	4.18	3.22905	77.25	22.75	0.004285599	yes	yes
Brevoortia tyrannus	EMBT2	1	1	250 mM EDTA pH 10.0	95.2	131.7	0.722854973	28	20.23993926	1.26	1.81	0.87	53.4	25.64268	48.02	51.98	0.137141321	yes	yes
Brevoortia tyrannus	EMBT6	2	1	250 mM EDTA pH 10.0	90.6	125.5	0.721912351	26.6	19.20286853	1.041	1.8	0.75	6.11	2.921802	47.82	52.18	0.016602717	yes	yes
Brevoortia tyrannus	EMBT10	3	1	250 mM EDTA pH 10.0	104.4	149.5	0.698327759	26.4	18.43585284	0.456	1.88	1.07	30.4	5.42032	17.83	82.17	0.135495115	yes	yes
Brevoortia tyrannus	EMBT14	4	1	250 mM EDTA pH 10.0	104.1	151.3	0.688037013	26.6	18.30178453	1.041	1.9	1.72	65.7	14.88762	22.66	77.34	0.277636205	yes	yes
Brevoortia tyrannus	EMBT18	5	1	250 mM EDTA pH 10.0	99.1	144.5	0.685813149	25.4	17.41965398	1.02	1.58	0.6	14.9	3.71457	24.93	75.07	0.064211551	yes	yes
Brevoortia tyrannus	EMBT22	6	1	250 mM EDTA pH 10.0	99	118.7	0.834035383	20.3	16.93091828	0.997	1.88	0.98	44.2	18.83362	42.61	57.39	0.149822825	yes	yes
Brevoortia tyrannus	EMBT3	1	2	95% EtOH	106.8	102.5	1.04195122	25.3	26.36136585	0.876	1.73	0.51	21	14.1015	67.15	32.85	0.026168978	yes	yes
Brevoortia tyrannus	EMBT7	2	2	95% EtOH	106.6	69.6	1.531609195	23.2	35.53333333	0.548	1.74	0.57	21.6	9.36576	43.36	56.64	0.034430319	yes	yes
Brevoortia tyrannus	EMBT11	3	2	95% EtOH	106.7	63.8	1.672413793	28	46.82758621	0.424	1.79	0.51	17.9	8.5741	47.9	52.1	0.019915398	yes	yes
Brevoortia tyrannus	EMBT15	4	2	95% EtOH	99	61.3	1.615008157	28.1	45.3817292	0.147	2.02	0.32	10.8	5.18184	47.98	52.02	0.012379784	yes	yes
Brevoortia tyrannus	EMBT19	5	2	95% EtOH	94	60.6	1.551155116	23.8	36.91749175	0.215	1.92	0.34	7.79	4.106109	52.71	47.29	0.009978714	yes	yes
Brevoortia tyrannus	EMBT23	6	2	95% EtOH	108.3	83.6	1.295454545	25.3	32.775	0.998	1.72	0.5	8.98	4.774666	53.17	46.83	0.01283092	yes	yes
Brevoortia tyrannus	EMBT1	1	3	Frozen, No preservative	102.3	101.9	1.003925417	28.4	28.51148184	1.018	1.62	0.49	15.4	8.37298	54.37	45.63	0.024646281	yes	yes
Brevoortia tyrannus	EMBT5	2	3	Frozen, No preservative	93.5	92.4	1.011904762	26	26.30952381	0.762	1.9	0.71	30.5	9.5221	31.22	68.78	0.079735005	yes	yes
Brevoortia tyrannus	EMBT9	3	3	Frozen, No preservative	100.5	99.6	1.009036145	30.1	30.37198795	1.125	1.79	0.9	64	19.392	30.3	69.7	0.146872177	yes	no
Brevoortia tyrannus	EMBT13	4	3	Frozen, No preservative	101	99.3	1.017119839	27.8	28.27593152	0.424	1.87	1.06	78.6	28.0602	35.7	64.3	0.178737878	yes	yes
Brevoortia tyrannus	EMBT17	5	3	Frozen, No preservative	90.9	90	1.01	28.7	28.987	1.459	1.82	1.19	132	47.8368	36.24	63.76	0.290348087	yes	yes
Brevoortia tyrannus	EMBT21	6	3	Frozen, No preservative	99.9	102.4	0.975585938	25.7	25.07255859	1.828	1.8	1.32	78.5	33.4881	42.66	57.34	0.179526552	yes	yes
Cynoscion regalis	EMCR2	1	1	250 mM EDTA pH 10.0	94	242.2	0.388109001	21.5	8.344343518	0.016	-0.48	-0.09	11.8	3.79488	32.16	67.84	0.095934689	yes	yes
Cynoscion regalis	EMCR6	2	1	250 mM EDTA pH 10.0	103.3	258.6	0.399458623	27.9	11.14489559	0.002	-0.07	0	8.31	2.928444	35.24	64.76	0.048287182	yes	yes
Cynoscion regalis	EMCR10	3	1	250 mM EDTA pH 10.0	90	204.2	0.440744368	22.6	9.960822723	0.113	1.79	0.67	49.2	28.35888	57.64	42.36	0.20923091	yes	yes
Cynoscion regalis	EMCR3	1	2	95% EtOH	107.5	79.3	1.355611602	21	28.46784363	0.342	2.09	-11.72	32.7	24.0672	73.6	26.4	0.030324742	yes	yes
Cynoscion regalis	EMCR7	2	2	95% EtOH	96.4	70.1	1.375178317	25.3	34.79201141	0.89	1.97	0.93	33.4	31.31918	93.77	6.23	0.005980741	yes	yes

Species	Sample ID	Specimen	Replicate	Treatment	Initial Sample Weight (mg)	Post-treatment Sample Weight (mg)	Correction Ratio	Weight of Tissue Used for Extraction (mg)	Corrected Weight of Tissue Used for Extraction (mg)	A_{260}	A_{260}/A_{280} Ratio	A_{260}/A_{230} Ratio	Total DNA Concentration (ng/µL)	LMW DNA Concentration (ng/µL)	%LMW	%HMW	nY (µg DNA/mg tissue)	*COI* PCR Amplification Success	*COI* Sequencing Success
Cynoscion regalis	EMCR11	3	2	95% EtOH	97.4	80.5	1.209937888	27.8	33.63627329	1.899	1.91	3.59	58.3	54.6271	93.7	6.3	0.010919462	yes	yes
Cynoscion regalis	EMCR1	1	3	Frozen, No preservative	95.7	92.8	1.03125	21.7	22.378125	0.732	1.93	-5.72	43.3	38.21658	88.26	11.74	0.022716023	yes	yes
Cynoscion regalis	EMCR5	2	3	Frozen, No preservative	97.1	93.7	1.036286019	25.9	26.8398079	-0.024	0.37	0.08	7.93	6.565247	82.79	17.21	0.005084809	yes	yes
Cynoscion regalis	EMCR9	3	3	Frozen, No preservative	105.3	93.9	1.121405751	20.8	23.32523962	-0.071	1.21	0.19	6.4	4.98176	77.84	22.16	0.006080281	yes	yes
Peprilus triacanthus	EMPT2	1	1	250 mM EDTA pH 10.0	101.9	205.3	0.49634681	24.9	12.35903556	-0.051	0.83	0.09	3.89	2.822195	72.55	27.45	0.008639873	yes	yes
Peprilus triacanthus	EMPT6	2	1	250 mM EDTA pH 10.0	107.6	191.9	0.560708702	22.4	12.55987493	-0.069	1.2	0.11	8.04	3.611568	44.92	55.08	0.035258568	yes	yes
Peprilus triacanthus	EMPT10	3	1	250 mM EDTA pH 10.0	103.3	189.2	0.545983087	24.9	13.59497886	-0.018	0.78	0.03	9.27	3.733956	40.28	59.72	0.04072124	yes	yes
Peprilus triacanthus	EMPT3	1	2	95% EtOH	106	87.8	1.207289294	28.8	34.76993166	0.272	1.79	0.33	6.92	5.24882	75.85	24.15	0.004806394	yes	yes
Peprilus triacanthus	EMPT7	2	2	95% EtOH	106.2	98.6	1.077079108	20.1	21.64929006	0.032	1.11	-0.11	7.31	4.973724	68.04	31.96	0.010791467	yes	yes
Peprilus triacanthus	EMPT11	3	2	95% EtOH	103.1	81.7	1.261933905	27.6	34.82937576	0.372	1.7	0.5	24.6	20.96412	85.22	14.78	0.010439119	yes	yes
Peprilus triacanthus	EMPT1	1	3	Frozen, No preservative	108.5	103.7	1.046287367	21.8	22.80906461	-0.026	0.86	0.04	11.6	10.77524	92.89	7.11	0.003615931	yes	yes
Peprilus triacanthus	EMPT5	2	3	Frozen, No preservative	93.9	90.1	1.042175361	26.1	27.20077691	0.046	4.45	-0.12	11.3	10.71918	94.86	5.14	0.002135307	yes	yes
Peprilus triacanthus	EMPT9-2	3	3	Frozen, No preservative	97.3	80.7	1.205700124	27.8	33.51846344	0.12	1.81	14.89	6.48	5.226768	80.66	19.34	0.00373893	yes	yes
Scomberomorus maculatus	EMSM2	1	1	250 mM EDTA pH 10.0	106.8	215.3	0.49605202	26.6	13.19498374	0.196	1.93	-4.6	11	3.795	34.5	65.5	0.054604084	yes	yes
Scomberomorus maculatus	EMSM6	2	1	250 mM EDTA pH 10.0	102.5	220.5	0.464852608	29.8	13.85260771	0.077	33.89	-0.3	5.81	2.533741	43.61	56.39	0.023650847	yes	yes
Scomberomorus maculatus	EMSM10	3	1	250 mM EDTA pH 10.0	96	171.7	0.559114735	22	12.30052417	0.049	15.18	-0.25	10.3	3.48243	33.81	66.19	0.055425036	yes	yes
Scomberomorus maculatus	EMSM14	4	1	250 mM EDTA pH 10.0	106.5	223.6	0.476296959	26.1	12.43135063	0.688	1.89	1.16	33.2	5.01652	15.11	84.89	0.226712936	yes	yes
Scomberomorus maculatus	EMSM18	5	1	250 mM EDTA pH 10.0	101	218	0.463302752	23.3	10.79495413	0.273	1.81	0.53	15.9	3.10527	19.53	80.47	0.118525098	yes	yes
Scomberomorus maculatus	EMSM22	6	1	250 mM EDTA pH 10.0	107.9	230.3	0.468519323	22.4	10.49483283	0.248	1.97	0.97	14.6	3.76388	25.78	74.22	0.103251954	yes	yes
Scomberomorus maculatus	EMSM3	1	2	95% EtOH	109	81.5	1.337423313	22.1	29.55705521	0.693	1.96	0.96	9.17	8.432732	91.96	8.04	0.002494389	yes	yes
Scomberomorus maculatus	EMSM7	2	2	95% EtOH	107.4	73.4	1.463215259	26.8	39.21416894	1.628	1.85	1.07	50.6	46.805	92.5	7.5	0.009677624	yes	yes
Scomberomorus maculatus	EMSM11	3	2	95% EtOH	104.6	79.7	1.312421581	23.7	31.10439147	0.6	2.19	1.19	43.7	40.93379	93.67	6.33	0.00889331	yes	yes
Scomberomorus maculatus	EMSM15	4	2	95% EtOH	100.8	69.5	1.450359712	25.2	36.54906475	1.462	1.88	1.41	74.8	62.88436	84.07	15.93	0.032601764	yes	yes
Scomberomorus maculatus	EMSM19	5	2	95% EtOH	106.2	80	1.3275	26.1	34.64775	1.692	1.85	1.16	69.5	59.1306	85.08	14.92	0.029928062	yes	yes
Scomberomorus maculatus	EMSM23	6	2	95% EtOH	105.9	86.9	1.218642117	21.1	25.71334868	0.66	1.84	0.97	40.2	29.93694	74.47	25.53	0.039913354	yes	yes
Scomberomorus maculatus	EMSM1	1	3	Frozen, No preservative	109.5	105	1.042857143	27	28.15714286	0.159	2.56	1.57	8.01	6.425622	80.22	19.78	0.005626913	yes	yes
Scomberomorus maculatus	EMSM5	2	3	Frozen, No preservative	101.1	95.2	1.06197479	23.5	24.95640756	0.11	4.69	3.79	15.7	11.66196	74.28	25.72	0.016180374	yes	yes
Scomberomorus maculatus	EMSM9	3	3	Frozen, No preservative	106.7	102	1.046078431	28.2	29.49941176	1.303	1.95	1.89	38.3	35.31643	92.21	7.79	0.010113998	yes	yes
Scomberomorus maculatus	EMSM13	4	3	Frozen, No preservative	100.6	101	0.996039604	22.4	22.31128713	0.269	1.89	0.66	16	5.7552	35.97	64.03	0.045917566	yes	yes
Scomberomorus maculatus	EMSM17	5	3	Frozen, No preservative	110.2	108.8	1.012867647	25.2	25.52426471	1.475	1.92	1.65	59.6	47.00056	78.86	21.14	0.049362597	yes	yes
Scomberomorus maculatus	EMSM21	6	3	Frozen, No preservative	103	101.9	1.010794897	22.3	22.5407262	0.264	1.81	1.04	18.2	10.1283	55.65	44.35	0.035809405	yes	yes
Trinectes maculatus	EMTM2	1	1	250 mM EDTA pH 10.0	96.5	153.7	0.627846454	25.1	15.758946	0.124	1.74	-0.66	9.06	3.494442	38.57	61.43	0.035316816	yes	no
Trinectes maculatus	EMTM6	2	1	250 mM EDTA pH 10.0	101.4	169	0.6	23.1	13.86	0.042	8.43	-0.28	8.64	3.439584	39.81	60.19	0.037521039	yes	no
Trinectes maculatus	EMTM10	3	1	250 mM EDTA pH 10.0	72.6	142.8	0.508403361	25	12.71008403	-0.025	0.71	0.05	9.99	3.558438	35.62	64.38	0.050602042	yes	no
Trinectes maculatus	EMTM14	4	1	250 mM EDTA pH 10.0	104.6	177.5	0.589295775	23.5	13.8484507	0.101	2.11	0.38	8.83	3.085202	34.94	65.06	0.041483326	no	no
Trinectes maculatus	EMTM3	1	2	95% EtOH	99.6	78.6	1.267175573	26.1	33.07328244	0.085	1.48	0.17	5.78	5.199688	89.96	10.04	0.001754625	no	no
Trinectes maculatus	EMTM7	2	2	95% EtOH	106.2	79.6	1.334170854	20.5	27.35050251	-0.014	0.43	-0.06	5.12	4.597248	89.79	10.21	0.001911307	no	no
Trinectes maculatus	EMTM11	3	2	95% EtOH	96	66.8	1.437125749	22	31.61676647	0.118	2.06	-0.87	12.5	9.58625	76.69	23.31	0.009215838	yes	no
Trinectes maculatus	EMTM15	4	2	95% EtOH	106.8	78.3	1.363984674	27.2	37.10038314	2.055	1.97	1.69	65.5	44.97885	68.67	31.33	0.055312502	no	no
Trinectes maculatus	EMTM1	1	3	Frozen, No preservative	103	102.2	1.007827789	23	23.18003914	-0.019	1.83	0.06	4.92	4.520988	91.89	8.11	0.00172136	no	no
Trinectes maculatus	EMTM5	2	3	Frozen, No preservative	103.7	106.9	0.970065482	29.6	28.71393826	-0.007	3.33	-0.04	4.66	4.334732	93.02	6.98	0.001132788	no	no
Trinectes maculatus	EMTM9	3	3	Frozen, No preservative	93.7	93.1	1.006444683	24.7	24.85918367	0.827	2.27	-425.82	5.87	5.542454	94.42	5.58	0.001317606	no	no
Trinectes maculatus	EMTM13	4	3	Frozen, No preservative	92.2	90.9	1.01430143	27.8	28.19757976	1.256	1.9	1.51	30.9	19.30632	62.48	37.52	0.041115869	no	no
